# Metabolomics Analysis Reveals the Participation of Efflux Pumps and Ornithine in the Response of *Pseudomonas putida* DOT-T1E Cells to Challenge with Propranolol

**DOI:** 10.1371/journal.pone.0156509

**Published:** 2016-06-22

**Authors:** Ali Sayqal, Yun Xu, Drupad K. Trivedi, Najla AlMasoud, David I. Ellis, Nicholas J. W. Rattray, Royston Goodacre

**Affiliations:** Manchester Institute of Biotechnology, School of Chemistry, The University of Manchester, Manchester, M1 7DN, United Kingdom; University of Cambridge, UNITED KINGDOM

## Abstract

Efflux pumps are critically important membrane components that play a crucial role in strain tolerance in *Pseudomonas putida* to antibiotics and aromatic hydrocarbons that result in these toxicants being expelled from the bacteria. Here, the effect of propranolol on *P*. *putida* was examined by sudden addition of 0.2, 0.4 and 0.6 mg mL^-1^ of this β-blocker to several strains of *P*. *putida*, including the wild type DOT-T1E and the efflux pump knockout mutants DOT-T1E-PS28 and DOT-T1E-18. Bacterial viability measurements reveal that the efflux pump TtgABC plays a more important role than the TtgGHI pump in strain tolerance to propranolol. Mid-infrared (MIR) spectroscopy was then used as a rapid, high-throughput screening tool to investigate any phenotypic changes resulting from exposure to varying levels of propranolol. Multivariate statistical analysis of these MIR data revealed gradient trends in resultant ordination scores plots, which were related to the concentration of propranolol. MIR illustrated phenotypic changes associated with the presence of this drug within the cell that could be assigned to significant changes that occurred within the bacterial protein components. To complement this phenotypic fingerprinting approach metabolic profiling was performed using gas chromatography mass spectrometry (GC-MS) to identify metabolites of interest during the growth of bacteria following toxic perturbation with the same concentration levels of propranolol. Metabolic profiling revealed that ornithine, which was only produced by *P*. *putida* cells in the presence of propranolol, presents itself as a major metabolic feature that has important functions in propranolol stress tolerance mechanisms within this highly significant and environmentally relevant species of bacteria.

## Introduction

Active pharmaceutical compounds (APCs), in their original states or their metabolites, are ubiquitous in the environment [1], and the levels of APCs in the aquatic ecosystems (e.g., lakes, rivers, seawater and estuaries) are a growing concern [2]. Pharmaceuticals are not only being introduced into the environment after consumption, but also via the disposal of unused or expired pharmaceuticals [3]. The levels of many pharmaceuticals in sewage treatment plants (STPs) have been detected at low concentrations in the range of ng L^-1^ to μg L^-1^ [1, 4–6]. A study in the United Kingdom revealed that the β-blocker propranolol is widely used, and for instance, around 12 tonnes of propranolol are consumed each year [4, 6, 7]. In addition, Ashton and co-workers (2004) showed that the presence of the β-blocker propranolol in STP effluents was highly likely at 76 ng L^-1^ (median level) [4].

Despite the fact that APCs are designed to have specific modes of action in the organism they were designed for, similar targets might control different metabolic processes in different species for which the original APC was not designed for [8]. In addition, the modes of action of the drugs within microbial systems are not fully understood. Thus, we and others believe it is necessary to increase our knowledge of the biological effects and fate of pharmaceuticals on microorganisms in the environment to appreciate the risks [9–11].

Indeed, bacterial communities inhabiting the benthic environment of riverbeds can be exposed to higher levels of APCs than expected, as it is known that these compounds can become concentrated in these areas [12–14]. Additionally, pharmaceuticals tend to bioaccumulate and induce impacts in aquatic and terrestrial environments due to their intrinsic pharmacokinetic properties [12]. A major adverse side effect of the presence of APCs in the environment is an increase in antimicrobial resistance that poses huge potential risk for the future, making the treatment of infections very difficult to cure, and there are several studies that have eloquently described the link between exposure to effluent and antimicrobial resistance [15–18].

Bacteria can adapt the activity of toxic substances by the employment of several resistant mechanisms including altering lipid composition, energy production, efflux pumps as well as other processes [19–22]. Efflux pumps, which transport toxic chemicals (usually waste products from normal metabolism) from the bacterial cell into the extra-cellular environment, are probably the most highly significant process which plays an important role in bacterial tolerance. One of these mechanisms is controlled by the ATP-binding cassette (ABC) transporters via the hydrolysis of ATP, whereas the transmembrane electrochemical gradient, particularly the proton motive force, is used by secondary transporters in order to drive drug efflux [23, 24]. In *Pseudomonas putida* DOT-T1E cells, three efflux pumps, which are genome-encoded, have been identified, and are termed TtgABC, TtgDEF, and TtgGHI. The TtgABC and TtgGHI pumps remove both organic solvents and some antibiotics, whereas the TtgDEF pump has been shown to be induced only by aromatic hydrocarbons [25–27].

Many studies have found that an enormous number of multidrug resistance (MDR) transport proteins are involved in the export of a wide range of antimicrobial compounds [23, 24, 28]. In *Pseudomonas* species, various studies linked solvent and antibiotic tolerance to the action of several efflux pumps [22, 25, 29, 30]. Moreover, solvent-tolerant microorganisms (e.g. *P*. *putida* DOT-T1E) play a crucial role in several biotechnological applications such as bioremediation, biocatalysis and agriculture [31–34]. Thus, an understanding of bacterial tolerant mechanisms is very important, in order to enhance the resistant systems for non-pathogenic strains and create altered strains with superior tolerance characteristics for industrial bioprocessing.

The qualitative and quantitative measurements of the metabolome of an organism can reveal its biochemical status and these data can be used to monitor and determine the function of genes [35, 36]. Metabolomics enables the identification and quantification of endogenous biochemical reaction products of cellular regulatory pathways and metabolite levels can be regarded as the ultimate response of biological system to environmental alterations and/or genetic factors. Metabolome analysis provides relevant information about specific cell types under different conditions that is important for a more holistic understanding of cell functions and properties [35]. A comprehensive assessment of the alteration in the metabolite levels in *P*. *putida* strains can be acquired using a combination of metabolic profiling and multivariate data analysis approaches. The interpretation of metabolic data is complicated, thus a wide range of different analytical strategies have been employed to measure the metabolome [37, 38]. By understanding metabolomics data the effect of stress on lowest molecular levels is revealed. This enables better understanding of altering metabolic pathways that are directly affected by change in bacterial genome.

In order to investigate the effects of propranolol on biological system, we have employed Fourier-transform infrared (FT-IR) spectroscopy to acquire metabolic fingerprints [39–41]. FT-IR spectroscopy involves the observation of bond vibrations from within molecules when a sample is excited by a beam from the mid-infrared region of the electromagnetic spectrum. Briefly, the infrared beam is transmitted through or reflected from a sample, with some of the infrared radiation being absorbed at particular wavelengths within the sample, and the remainder continuing on to a detector, before being Fourier transformed and analysed via a computer. This results in an infrared absorbance spectrum which can be referred to as a metabolic “fingerprint” as it is characteristic of any chemical or biochemical substance. The fundamentals of FT-IR have been described in far greater detail elsewhere [40, 42] but its main advantages are that it is very rapid (taking seconds per sample), high-throughput, with 96 and 384 well sampling plates, reagentless, and non-destructive. FT-IR has been applied to a very wide-range of biological studies including clinical [41, 43] and microbiological [44] analyses since the very early 1990s when Dieter Naumann and co-workers demonstrated its potential use for bacterial characterization [45]. Metabolic profiling approaches are powerful in that in contrast to FT-IR spectroscopy they can be used to identify, quantify and detect the metabolites within the biological system, and gas chromatography mass spectrometry (GC-MS) is currently a very popular method for analyzing central carbon and nitrogen metabolism [46–48]. Changes identified in the metabolome can be considered to be hypothesis generating and as such can inform our biochemical knowledge [49, 50]. With respect to bacterial strain tolerance we believe that the observed metabolite changes can prove to be indicative of novel adaption mechanisms or may support postulated adaption mechanisms for which there is little evidence up to date.

The aim of this study was to investigate the changes in metabolite levels within *P*. *putida* DOT-T1E strains in the presence and absence of propranolol and determine if these changes were associated to efflux pumps or other adaptation mechanisms within these bacteria. To enable this, FT-IR spectroscopy was utilised as a rapid, high-throughput screening tool in order to identify phenotypic alterations in bacterial cultures exposed to propranolol, and metabolic profiling using GC-MS was employed to examine the change in metabolites at specific time points before and after challenge with propranolol.

## Material and Methods

### Bacterial Strains and Cultivation of Bacteria

Three bacterial strains of *P*. *putida* DOT-T1E were used in this study, their relevant characteristics, and references for further information on each strain are listed in Table 1. All strains were sub-cultured in triplicate to obtain axenic cultures. Individual colonies were then picked and transferred from plates into 250 mL flasks containing 50 mL of autoclaved Lysogeny broth (LB) medium and incubated at 24 h at 30°C in an orbital incubator (Infors HT Ltd, UK) shaking at 200 rpm.

**Table 1:** Bacteria used in this study.

Bacteria	Relevant characteristics^a^	Reference
***P*. *putida* DOT-T1E**	Ap^r^ Rif^r^ Tol^r^	[84]
***P*. *putida* DOT-T1E-PS28**	Rif^r^ Sm^r^ *ttgH*::VSm	[27]
***P*. *putida* DOT-T1E-18**	Rif^r^ Km^r^ *ttgB*::´*phoA*-Km	[25]

^a^ Resistance to Ap^r^: ampicillin, Rif^r^: rifampin, Sm^r^: streptomycin, Km^r^: kanamycin and Tol^r^: toluene

### Growth Curve Monitoring

Bacterial growth curves were monitored manually using an orbital incubator and UV instrument at 660 nm (Biomate 5, CarePlanTM, UK). All samples were normalised to an optical density (OD) of 0.02 in 250 mL flasks containing 50 mL LB medium. *P*. *putida* DOT-T1E cultures were incubated at 30°C and 200 rpm. During the 24 h time course of, 100 μL samples were taken at various time points (0, 2, 4, 6, 8, 10, 12 and 24 h) for OD measurement.

### Growth in Response to Propranolol Shock, Sample Collection and Analysis

Cells were grown in 50 mL of LB medium for 5 h at 30°C and 200 rpm. Once cell cultures reached the mid-exponential phase, samples were divided into two groups. One group was kept as a control, and to the second group propranolol was added at three different concentrations (0.2, 0.4 and 0.6 mg mL^-1^). These cultures were then incubated for an additional 8 h.

#### Growth curve measurement

At various time points (0, 1, 3, 5, 7, 9, 11 and 13 h) before and after the addition of propranolol, a 100 μL sample was taken for OD measurement. Growth was recorded as an increase or decrease in turbidity at 660 nm. This work was undertaken in biological triplicates.

#### FT-IR sample collection

After 60 min of the addition of propranolol, an aliquot (2 mL) sample was transferred to 2 mL tube, and the ODs of the samples were recorded for normalisation. All measurements were performed in triplicate.

#### Sample preparation for FT-IR spectroscopy

An aliquot (2 mL) sample from each flask was transferred to 2 mL tube and centrifuged at 11500 ×*g* for 5 min at 4°C. The supernatant was removed and discarded, and the remaining pellet was washed twice with 2 mL of physiological saline solution (0.9% NaCl) and centrifuged (11500 ×*g*, 5 min, 4°C) and the supernatant discarded. The remaining cell pellets were stored at -80°C until required.

A 96-well silicon FT-IR plate (Bruker Optics, Banner Lane, Coventry, UK) was cleaned with 5% sodium dodecyl sulfate (SDS) and rinsed with deionised water and allowed to dry at room temperature. Cell pellets were then removed from -80°C and allowed to thaw on ice. Samples were normalised according to OD at 660 nm and resuspended in physiological saline and gently vortexed. Aliquots (20 μL) of each sample were randomized and spotted in triplicate onto a silicon FT-IR plate. The prepared plates were then dried on a desiccator at ambient temperature for 7 h. This step was applied to minimise any signal arising from water absorbance in the mid-IR region.

#### FT-IR setup

The prepared silicon sample plate was loaded onto a motorised microplate module HTS-XT^™^ under the control of a PC programmed with OPUS software version 4. Spectra were acquired using a Bruker Equinox 55 FT-IR spectrometer (Bruker Optics, Banner Lane, Coventry, UK) in transmission mode as described previously [51], with a deuterated triglycine sulfate (DTGS) detector over the wavenumber range 4000–600 cm^-1^, with a resolution of 4 cm^-1^, 64 scans were co-added and averaged in order to improve the signal-to-noise ratio. Three technical replicates were obtained from each sample, and a total of 324 spectra were collected.

#### FT-IR data analysis

FT-IR data were converted to ASCII format using OPUS reader software and analysed using Matlab version 2012 (MathWorks, Natick, MA). All FT-IR spectra were CO_2_ corrected by replacing the region from 2400 to 2275 cm^-1^ with a linear trend and then scaled using extended multiplicative signal correction (EMSC) [52].

Statistical analysis of the preprocessed data was performed using principal component analysis (PCA) [53] and discriminant function analysis (DFA). PCA was used to generate set of latent variables (PCs) which retain the major variance of the data whilst decreasing the dimensionality; DFA was then used to create a set of discriminant functions (DFs) based on PCs which maximize the differences between the known groups (classes) [54, 55]. PC-DFA was performed using 10 PCs and 3 DFs, and the class structure for the DFA algorithm was based on the biological replicates of samples of the same conditions.

#### GC-MS sample collection

15 mL samples were quenched at three time points 0, 10 and 60 min before and after the addition of propranolol (0 min refers to the point immediately before the addition of propranolol). This procedure was performed with four biological replicates.

#### Metabolic quenching and metabolite extraction

Generally, a rapid inactivation of metabolism is achieved by alteration in pH or temperature [56]. Thus, in order to halt metabolism culture samples (15 mL) were plunged into a double volume of 60% cold methanol (-50°C) in a 50 mL tube. The quenched culture mixture was centrifuged (3000 ×*g*, 10 min, 1°C), and then the supernatant was discarded, while the cell pellets were stored at -80°C until required for metabolite extraction [57].

The biomass pellets were resuspended in 750 μL of freshly prepared cold methanol (80%). The solution was then transferred to a 2 mL Eppendorf tube. This was followed by a freeze-thaw cycle in order to extract the intracellular polar metabolites from the cells. Samples were centrifuged at (13500 ×*g*, 3 min, 4°C) and the supernatant was transferred to new tubes and stored on dry ice [57]. The extraction was performed again on the remaining pellet and both supernatants were combined and again stored on dry ice. A final aliquot (1400 μL) of metabolite extracts were normalised using 80% methanol according to OD at 660 nm. A quality control (QC) sample [58] was prepared by transferring 100 μL from each of the sample to a new (15 mL) centrifuge tube. This was followed by the addition of (100 μL) of internal standard solution (0.2 mg mL^-1^ glycine-*d*_*5*_, 0.2 mg mL^-1^ benzoic-*d*_*5*_ acid, 0.2 mg mL^-1^ lysine-*d*_*4*_, and 0.2 mg mL^-1^ succinic-*d*_*4*_ acid) to all samples. The samples were lyophilized for 16 h by speed vacuum concentrator (concentrator 5301; Eppendorf, Cambridge, UK), and then the pellet was stored at -80°C for further analysis.

#### GC-MS derivatization process

Samples were derivatized prior to GC-MS analysis in two stages as described previously by Wedge and co-workers [59]. The first step, (50 μL) of *O*-methoxylamine hydrochloride diluted in pyridine (20 mg mL^-1^) was added to the samples and then samples were heated on a heating block at 65°C for 40 min. The second step, (50 μL) of MSTFA (*N*-methyl-trimethylsilyltrifluoroacetamide) was added to the samples followed by heating for 40 min. At the end of second step, 20 μL of retention index was added. After each addition in all three steps described above samples were vortexed for 10 s and centrifuged at 13500 ×*g* for 15 min.

#### GC-MS instrument setup

Samples were randomised and analysed by gas chromatography electron ionisation time-of-flight mass spectrometry (GC-TOF-MS) using an Agilent 6890 GC instrument coupled to a LECO Pegasus III TOF mass spectrometer (Leco, St. Joseph, MI, USA), as described previously [59–61]. GC column (VF-17MS column, 0.25 mm ID × 30 m × 0.25 μm film thickness, Varian, cat. no. CP8982) was employed at a constant helium carrier gas flow of 1 mL min^-1^, with a temperature program starts at 70°C and end at 300°C. The mass spectrometer source is operated at a temperature of 250°C in electron ionization (EI) mode, with an electron energy of 70 eV and the detector is operated in the range 1400–1800 V. Raw data processing was undertaken using LECO ChromaTOF v3.26 in order to construct a data matrix of metabolite peak *vs*. sample and infilled with peak areas for metabolites that were detected. A reference database was prepared that contained retention times, quant mass, peak area, retention index value and peak number related to each peak by analysing QC samples. The identification of analytes was based on both spectral similarity and matched with retention indices. In-house library as well as NIST library was used for identification, and we followed MSI guidelines for metabolite identification [62].

#### GC-MS data analysis

For statistical analysis multi-block PCA [63] was used with three different types of blockings. The first type of blocking is strain | time×dosage blocking. This blocking partitioned the data into 9 blocks. Each block contained all the samples from the same time point with the same dosage of propranolol, e.g. all the samples with 0.2 mg mL^-1^ propranolol, collected at 0 min were assigned to one block, those with 0.4 mg mL^-1^ propranolol, collected at 10 min were assigned to another block and so on. Across different blocks, the strains were matched so that in every block the first 4 samples were *P*. *putida* DOT-T1E, the next 4 samples were *P*. *putida* DOT-T1E-18 and the last 4 samples were *P*. *putida* DOT-T1E-PS28. Based on the same principle, dosage | strain×time blocking partitioned the data into 6 blocks (samples at 0 min were not included for this type of blocking as this time point refers to the point immediately before the addition of propranolol), each block had the samples of the same strain and same time points, the dosage of propranolol were matched. Such blocking allowed MB-PCA to detect the effect of each of the factor of interest (i.e., strain, time and dosage of propranolol) separately without the inference from others [64].

A total of 200 features were detected by GC-MS. The natural logarithm (ln) was first applied on the peak area of the detected peaks. Data were mean centred and then auto-scaled then subjected to MB-PCA. The potentially most significant variables were identified by selecting the most predominant averaged block loadings. Finally, box-whisker plots were used to visualise the data. These analyses were conducted using in-house scripts under the Matlab 2014a (Mathworks, Natick, MA) environment. The data are available at MetaboLights (http://www.ebi.ac.uk/metabolights/): study identifier MTBLS320.

## Results and Discussion

### Characterization of *P*. *putida* DOT-T1E Strains

**Fig 1:**
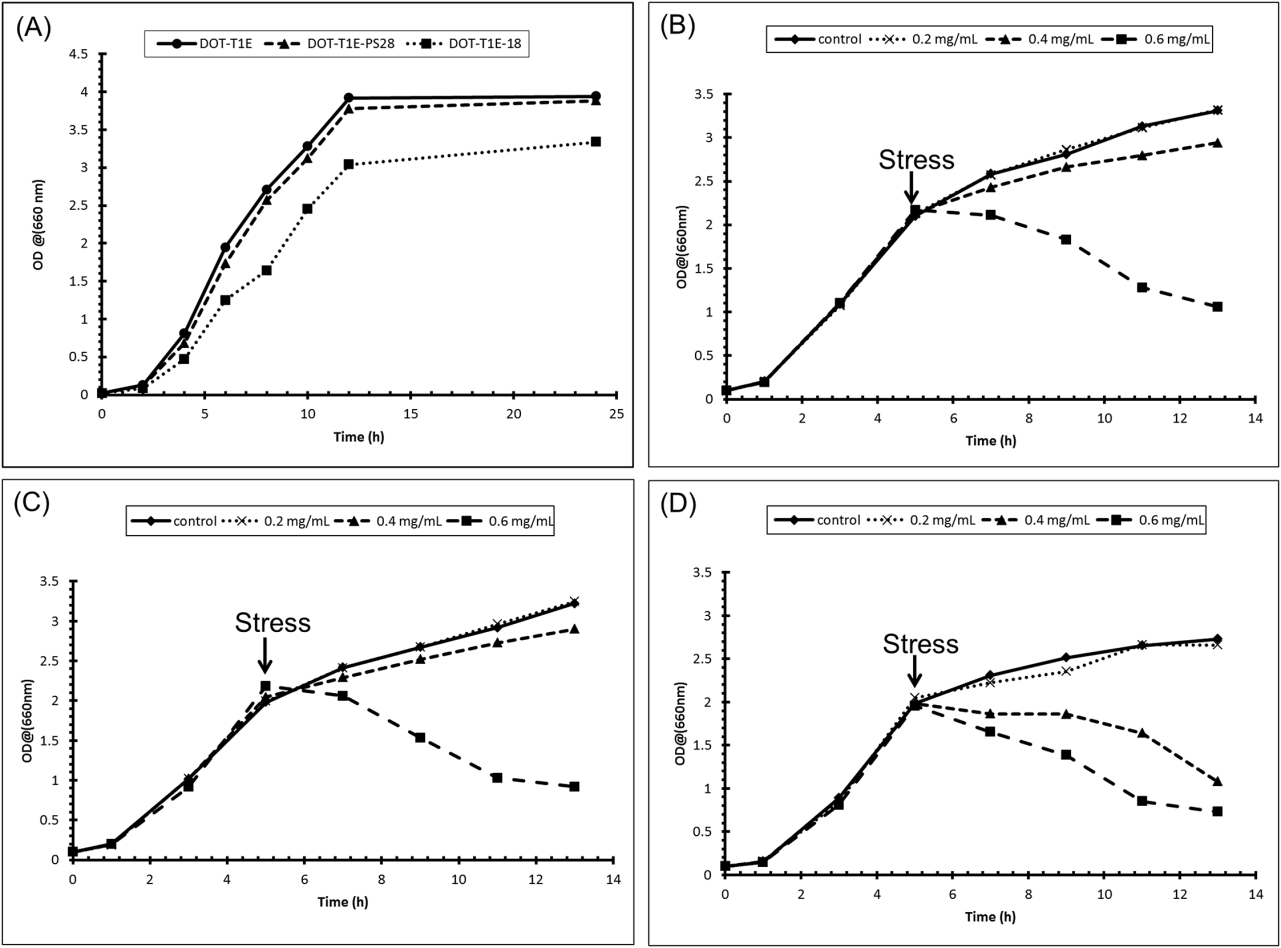
Growth curves of *P*. *putida* strains. (A) all three *P*. *putida* DOT-T1E strains in LB medium without propranolol; (B) *P*. *putida* DOT-T1E, (C) *P*. *putida* DOT-T1E-PS28, and (D) *P*. *putida* DOT-T1E-18 in the presence of propranolol. A 1/10 dilution of 100 μL samples were prepared for OD measurement at 660 nm.

Growth curve experiments were undertaken for *P*. *putida* strains to determine the optimum points to induce abiotic stress using propranolol. The resultant growth curves are displayed in (Fig 1A) and these show that there were no significant differences in the pattern of growth between the wild type DOT-T1E and the mutant DOT-T1E-PS28 (lacking the TtgGHI pump) over the 24 h incubation period. Whilst under the same conditions, the mutant DOT-T1E-18 (lacking the TtgABC pump) grew slightly poorly in comparison to the other strains. This result was in agreement with previous observations which show that *P*. *putida* DOT-T1E-PS28 grew on LB medium and had similar growth generation time to the wild type [27]. However, the mutant DOT-T1E-18 showed less growth compared to the wild-type and this could be a result of the waste products made during cellular metabolic processes accumulating to toxic levels due to the lack of TtgABC pump, resulting in slower growth. To be able to investigate the metabolome changes between the wild type and the two mutants, cells were cultured in the absence of propranolol, GC-MS analysis was performed and this was followed by chemometrics.

MB-PCA of all *P*. *putida* strains was carried out and the result showed an obvious clustering pattern as can be seen in (S1 Fig). It was clear from this analysis that *P*. *putida* DOT-T1E-18 was very different to the other two strains although weak separation can also be observed between *putida* DOT-T1E and *P*. *putida* DOT-T1E-PS28. In addition, MB-PCA loading plots were plotted in order to investigate the significant metabolites associated with the different growth behaviour. It can be seen that many metabolites were most abundant in *P*. *putida* DOT-T1E-18 and least abundant in *P*. *putida* DOT-T1E. Box-whisker plots were generated and these generally supported the increased metabolite levels in DOT-T1E-18 (S2 Fig). During the growth of the three *P*. *putida* strains, a number of metabolites detected by GC-MS were compared (e.g. carbon and nitrogen metabolism; *viz*., sugars, sugar phosphates, amino acids, organic acids).

A schematic summary of the detected metabolites by GC-MS of central metabolic pathways in *P*. *putida* DOT-T1E strains is shown in S3 Fig and S1 Table. It can be seen that the level of a total of 9 metabolites were similar in the mutant DOT-T1E-PS28 compare to the wild-type DOT-T1E, while only 3 metabolites had similar levels in the mutant *P*. *putida* DOT-T1E-18 in comparison to the wild type. These results would suggest that the TtgABC pump is involved in the removal of toxic metabolites produced during the log phase. In addition, the accumulation of toxic products might result in changes in the level of amino acids due to the activation of other metabolic pathways to deal with waste products.

### Characterization of *P*. *putida* DOT-T1E Strains to Propranolol Shocks

#### Minimal inhibitory concentration (MIC)

In order to study the effect of propranolol on *P*. *putida* DOT-T1E cultures, it was necessary to establish the MIC of each bacterial strain when cultured in LB media and challenged with different levels of propranolol and the results are recorded in S2 Table. The visible growth of the wild-type DOT-T1E, mutant DOT-T1E-PS28 and mutant DOT-T1E-18 were inhibited at 1.5, 1.5 and 0.8 mg mL^-1^ of propranolol respectively. The resistance of DOT-T1E-PS28 to propranolol was the same as the wild-type. However, it was reduced for the mutant DOT-T1E-18, suggesting that the extrusion of propranolol by the TtgABC pump could play a more crucial role than TtgGHI pump. Observations similar to these findings have been reported by Rojas and co-workers [27] testing MIC of several antibiotics for *P*. *putida* DOT-T1E strains, in which the DOT-T1E-18 mutant was more sensitive to those antibiotics than DOT-T1E. Nevertheless, the DOT-T1E-PS28 mutant showed similar sensitivity to the wild-type.

#### Bacterial growth in the presence of propranolol

From interpretation of the growth curves (Fig 1A), it was decided to induce propranolol stress after 5 h (once the cultures reached their mid-exponential phase) at three different concentrations of propranolol (0.2, 0.4 and 0.6 mg mL^-1^) below the MIC. The effect of propranolol on *P*. *putida* cells was then studied in liquid culture medium after cells had been pre-grown on LB liquid medium, and following challenge with propranolol. Growth curve results from *P*. *putida* cultures are shown in (Fig 1B–1D). In general, slight variations were noted in the growth patterns between *P*. *putida* DOT-T1E and DOT-T1E-PS28 species exposed to 0.2 and 0.4 mg mL^-1^ propranolol, though considerable effects on the same cultures were observed when cultures were exposed to 0.6 mg mL^-1^ propranolol across a 13 h growth period.

By contrast, a marked effect was observed in *P*. *putida* DOT-T1E-18 when exposed to 0.4 and 0.6 mg mL^-1^ concentrations of propranolol. Strain tolerance is an energy intensive process, and it was noted that the growth yields of *P*. *putida* DOT-T1E cultures in the presence of 0.6 mg mL^-1^ were reduced by five-fold compared to the control cultures. This decrease in the growth yield might result in consumption of energy by various mechanisms in order to protect the cells from further damage. One study examined the growth yields of *Pseudomonas* upon sub-lethal toluene dosages and it was found that the presence of toluene led to lower yields and that the growth yield reduced linearly with increasing toluene concentrations [65]. This report deduced that the decrease in yield associated with the presence of toluene could be due to energy-consuming adaptation mechanisms initiated to protect cells from excessive damage.

To assess bacterial membrane integrity during the growth of bacteria following propranolol perturbation a LIVE/DEAD *Bac*Light bacterial viability assay was used, and the green and red fluorescence emissions were measured using a Flexstation 3 Microplate Reader (Molecular Devices, USA). The ratio of green to red fluorescence and the percentage of live cells from TVC plates estimations in the *P*. *putida* suspension are shown (S3 Table). It was clear from these measurements that cell viability decreased linearly with increasing propranolol indicating the toxic effect of propranolol on *P*. *putida* DOT-T1E strains.

#### FT-IR fingerprinting of cell cultures

**Fig 2:**
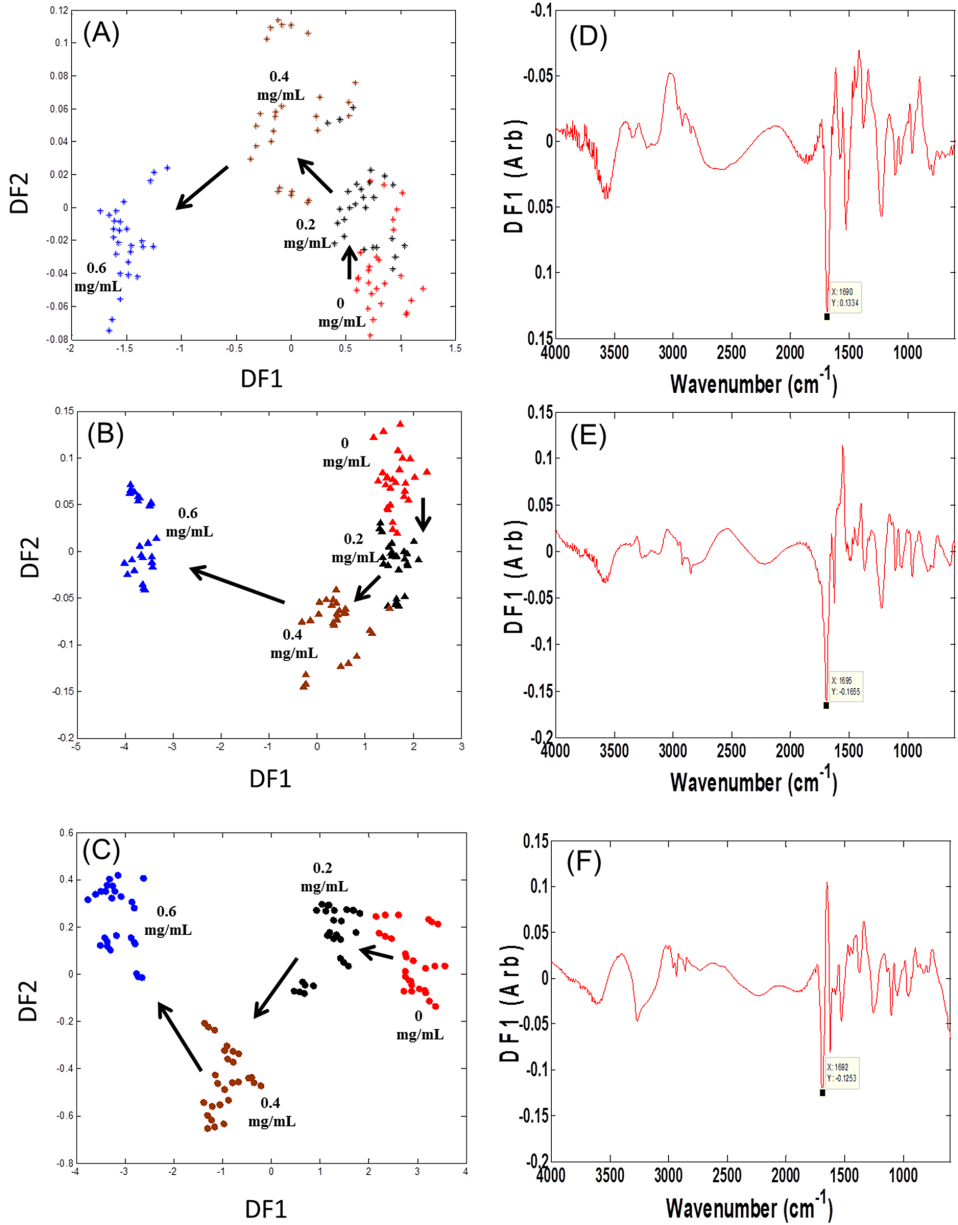
PC-DFA scores plots of FT-IR data for three different strains of *P*. *putida* strains upon propranolol shock. Symbols represent different strains. (A) *P*. *putida* DOT-T1E is the wild type (stars) and ten PCs with a total explained variance (TEV) of 99.43% were used for the DFA, (B) *P*. *putida* DOT-T1E-PS28 is the mutant (closed triangles) and ten PCs with a TEV of 99.65% were used for the DFA, (C) *P*. *putida* DOT-T1E-18 is the mutant (closed circles) and ten PCs with a TEV of 99.03% were used for the DFA. Colour coding: control with no propranolol (red), cells exposed to 0.2 mg mL^-1^ propranolol (black), 0.4 mg mL^-1^ propranolol (brown), and 0.6 mg mL^-1^ propranolol (blue). Arrows indicate the direction of shift because of the increase of propranolol concentration. (D) PC-DFA loadings plot for *P*. *putida* DOT-T1E. (E) PC-DFA loadings plot for *P*. *putida* DOT-T1E-PS28, (F) PC-DFA loadings plot for *P*. *putida* DOT-T1E-18. Significant loadings were assigned to bacterial proteins.

FT-IR spectroscopy was employed to investigate whether the phenotype of an organism had changed by exposing it to gradient levels of propranolol. PC-DFA scores plots were produced in order to visualise the distribution of samples based on their IR metabolic fingerprints (Fig 2A–2C). From inspection of the PC-DFA scores plots of the biomass samples, it was possible to determine that there was an obvious separation between the different culture conditions. There was also a clear trajectory based on concentration (annotated with arrows) with samples from control cultures following a trend from right to left across the plot space due to the increase of propranolol concentrations. This clustering pattern was anticipated and suggests that propranolol stress has had a clear additive effect on the bacterial cells and this is reflected in the FT-IR results. In other analyses these PC-DFA models were validated by test set projection (S4 Fig) and these ensure that the model quality is of a high standard, and that the obtained subsequent conclusions drawn from the data are valid and robust.

To assess the relevant metabolites causing these separations in PC-DFA scores plots, the loadings plots for the first discriminant functions were plotted (Fig 2D–2F). Multiple changes occur within these loadings plot with the largest variances being observed between wavenumbers 1700–1600 cm^-1^. In this region of the mid-infrared the majority of vibrational bands are associated with protein components of the sample; most notably amide I (C = O stretching at 1690–1620 cm^-1^) and amide II (combination of C-N stretching and N-H bending). These results suggest that the most significant effect over the duration of the 1 h incubation period following drug shock is associated with alterations to proteinaceous components of bacteria. The profile of proteins in different *P*. *putida* strains—T1E and S12—upon exposure to toluene has been investigated previously, and it was revealed that almost 90 proteins were up-regulated as a result of an exposure of strains to toluene in which some of these proteins relate to efflux pump systems [22, 66, 67]. Therefore, it is perhaps not surprising that the most significant changes observed from the interpretation of infrared spectra were in the vibrational frequency of the proteins components, and we can infer from this that some proteins were up-regulated to cope with the presence of propranolol.

#### GC-MS metabolic profiling of cell cultures

Recently, attention has been focused on studying the stress responses in bacteria employing metabolomics-based approaches [68–70], and this has involved a wide range of disciplines such as drug discovery, metabolic engineering and medical sciences [71–75]. In this study, we employed GC-MS to create metabolic profiles of bacterial stress to propranolol, as the knowledge of variations within the metabolome following chemical perturbation could lead to a more in-depth understanding of strain specific stress responses within these bacteria.

As there are multiple potentially interacting factors that we have in our experiment with respect to propranolol dose, bacterial strain, as well as time, MB-PCA was used for analysis. MB-PCA with dosage | strain×time blocking (see materials and methods) was undertaken and a gradient effect corresponding to differing dosages of propranolol can be seen on the resultant scores plot (S5 and S6 Figs). We observed nine metabolites that were differentially expressed between control and different dosages of propranolol and these were statistically significant. However, four metabolites (cystathionine, glutamine and two unknowns) decreased with increase in dosage of propranolol, four metabolites (ornithine, propranolol and two unknowns) increased with dosage whereas no clear pattern was seen for one metabolite (unknown).

**Fig 3:**
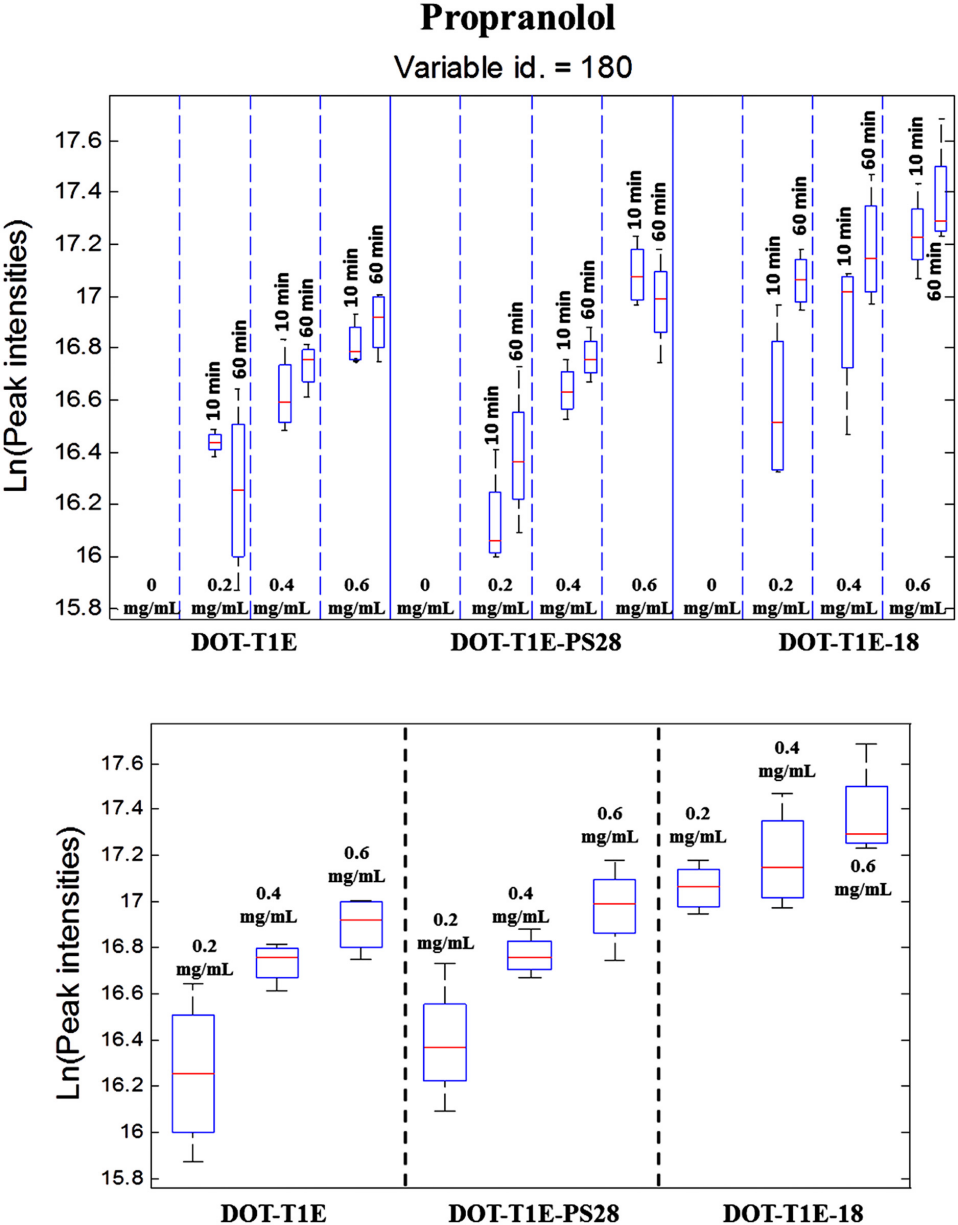
Box-whisker plots showing the changes in metabolite levels in control and cells exposed to propranolol for 4 *biological* replicates. Variable 180 was identified as propranolol. (Red line) indicates the median *m/z* intensity. (A) Represent the data for 3 *P*. *putida* strains, 4 concentrations of propranolol and 3 time points, dashed lines separate different concentration levels of propranolol and solid line separates different strains. (B) Represent the data for 3 *P*. *putida* strains, 3 concentrations of propranolol and 1 time point at 60 min, dashed lines separates different strains.

Interestingly, it was found that two of these metabolites (variables 180 and 100) were only detected following the exposure of *P*. *putida* strains to all three concentrations of propranolol groups but not in the control. Variable 180 was identified by an in-house database as propranolol itself, and Fig 3A shows that exposure of cells to propranolol resulted in the accumulation of propranolol in comparison to non-exposed cells. These data also show that the level of propranolol in *P*. *putida* stains were detected at both time points at 10 and 60 min, and it was noticed that the accumulation of propranolol in the exposed cells increased as the concentration of the propranolol increased.

In addition, comparing the level of propranolol between the wild-type and the mutants only at 60 min (Fig 3B), it was observed that *P*. *putida* DOT-T1E (wild-type) and *P*. *putida* DOT-T1E-PS28 (lacking TtgGHI pump) showed high similarities in the level of propranolol at all tested concentrations. By contrast, the amount of propranolol accumulating in the *P*. *putida* DOT-T1E-18 (lacking TtgABC pump) was higher than the other strains. This could be further evidence for the activity of efflux pump system in *P*. *putida* cells due to the presence of propranolol at different levels. In addition, these results would suggest that the TtgABC efflux pump is the main extrusion pump for propranolol and that it plays a more important role than the TtgGHI pump. These findings, which agree well with other studies, show that the TtgABC pump in *P*. *putida* DOT-T1E is the main antibiotic extrusion pump, and it has the ability to extrude flavonoids, tetracycline, chloramphenicol and ampicillin in addition to other solvents such as toluene [76–78].

**Fig 4:**
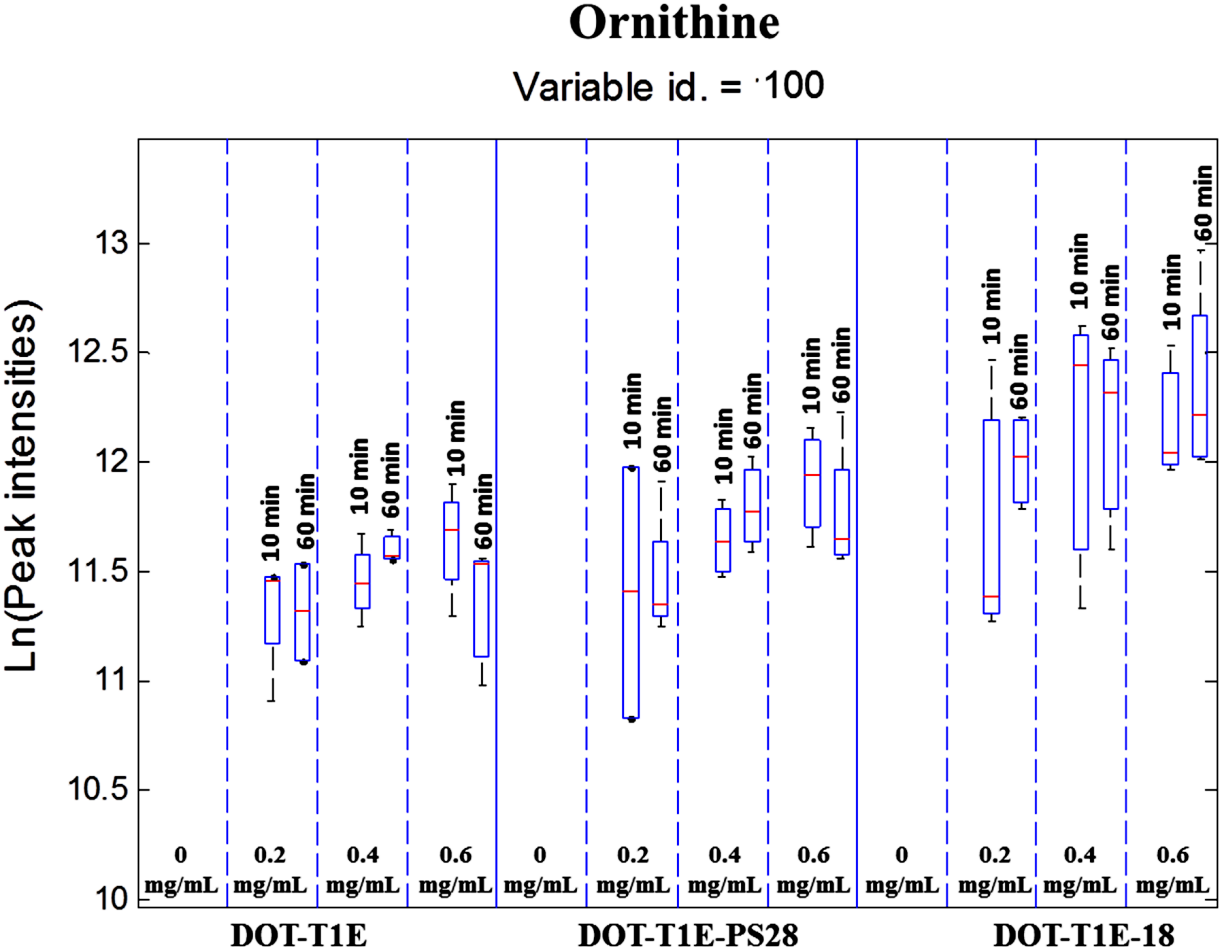
Box-whisker plot showing the changes in ornithine levels (variable id 100) in control and exposed cells to propranolol. (Red line) indicates the median *m/z* intensity. These plots represent the data for 3 *P*. *putida* strains, 4 concentrations of propranolol and 3 time points, for 4 biological replicates. Dashed lines separate different concentration levels of propranolol and solid line separates different strains.

Interestingly, the other significant variable, 100, was identified as ornithine (ChEBI ID 15729) again from an in-house library generate on the same instrument [62]. Ornithine production was detected within 10 min after exposure to propranolol, and the level of ornithine in the wild-type DOT-T1E and mutant DOT-T1E-PS28 shows an increase at 0.2 mg mL^-1^ propranolol and a further almost linear increase in the presence of 0.4 and 0.6 mg mL^-1^ propranolol (Fig 4). By contrast, the production of this metabolite in the mutant DOT-T1E-18 exhibits an increase at 0.2 mg mL^-1^ propranolol followed by a further increase at 0.4 mg mL^-1^ followed by a decrease toward 0.6 mg mL^-1^ propranolol. Furthermore, the level of ornithine was further decreased, from 10 to 60 min at 0.6 mg mL^-1^ propranolol for both *P*. *putida* DOT-T1E and DOT-T1E-PS28, while it was increased for *P*. *putida* DOT-T1E-18 under the same conditions. This metabolite is very important, as it is only produced by the *P*. *putida* cells in the presence of propranolol and our data suggest that this is linked to bacterial tolerance mechanisms, further studies are needed in order to understand this role and comprehend whether this is a cause or effect relationship.

In addition, in *P*. *putida* T1E and S12 proteomic analysis found that several proteins of the TCA cycle involved in energy production were up-regulated upon toluene exposure, indicating a requirement for enhanced metabolism and high energy demands because of toluene exposure in order to power efflux pumps that extrude solvent from the cells [22, 66], which is in agreement with several proteomics, and transcriptomics studies [66, 79]. The up-regulation of several terminal oxidase genes upon solvent stress in *P*. *putida* T1E suggests that demands on energy consumption are necessary to cope with the presence of solvents, in particular due to high activity of efflux pumps [80]. Ornithine can be synthesised via the TCA cycle in which glutamate is converted into ornithine, as previously reported for *P*. *putida* [81]. The production of ornithine in the presence of propranolol is interesting, as this observation would suggest that *P*. *putida* DOT-T1E may use this amino acid for energy production to power efflux pumps, or in order to activate other metabolic pathways that are important in bacterial tolerance to propranolol.

In addition, the primary building block of biological membranes mainly consists of glycerophospholipids such as phosphatidylglycerol (PG), phosphatidylethanolamine (PE) and cardiolipin (CL); however, other lipids classes (e.g. ornithine lipids) have been described as well, which contain a 3-hydroxy fatty acyl group attached in amide linkage to the α-amino group of ornithine. This lipid can be formed only by specific groups of bacteria or under certain stress conditions [82]; although these have not yet been reported in *P*. *putida*. It is possible that the ability to produce ornithine under propranolol stress in *P*. *putida* strains is linked to lipid production, however we have no direct evidence for this yet.

**Fig 5:**
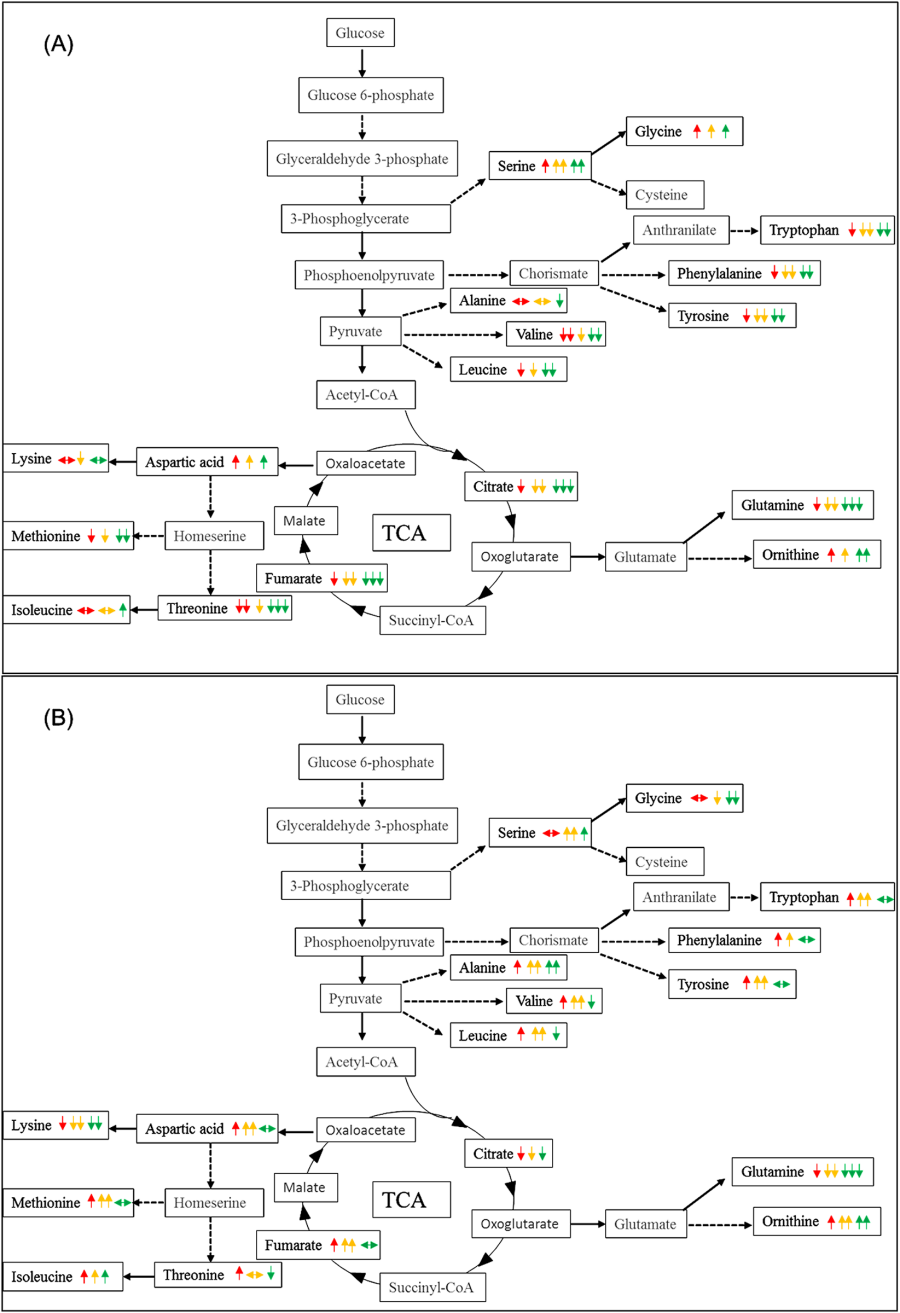
Schematic metabolic diagram of central carbon metabolism in *P*. *putida* DOT-T1E adapted to propranolol showing the level of metabolites for cells exposed to propranolol compared to the control. Metabolites were detected and identified by GC-MS. Metabolites indicated in black were observed, while metabolites indicated in grey were not detected. The median *m/z* intensity (red line) in the box- whisker plots was used to compare the level of metabolites. (A) Represent the level of metabolites at 10 min, while (B) the level of metabolite at 60 min. Traffic light system represents different concentration of propranolol. Red, yellow and green represent exposed cells to 0.2, 0.4 and 0.6 mg/mL of propranolol respectively. Up-arrow, down-arrow and steady arrow indicate an increase, a decrease and no change in the level of metabolite respectively. The number of arrows represents the level of metabolites. Slight change (single arrow), medium change (double arrows) and high change (triple arrows).

**Fig 6:**
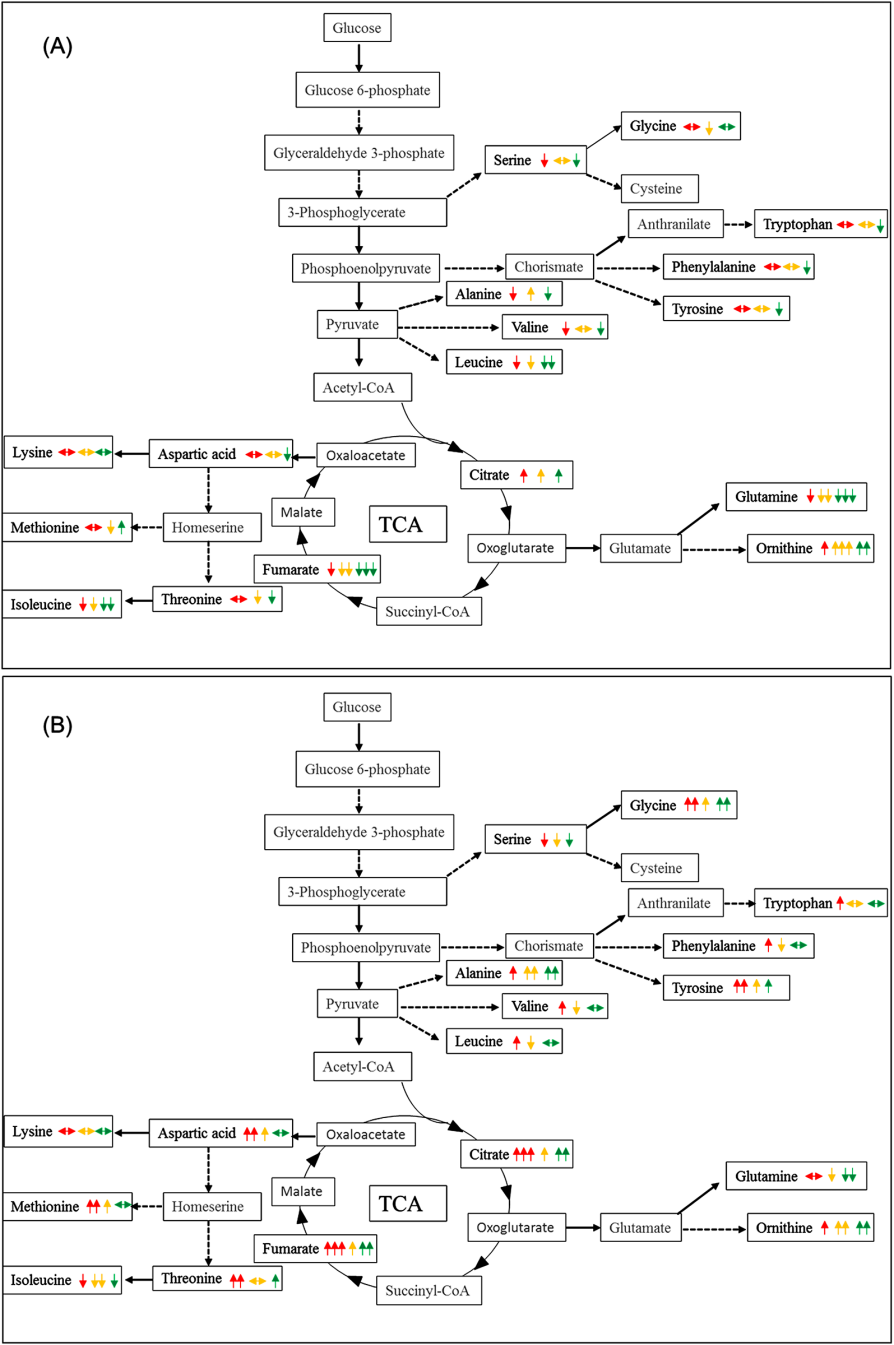
Schematic diagram of central carbon metabolism in *P*. *putida* DOT-T1E-18 adapted to propranolol showing the level of metabolites for cells exposed to propranolol compared to the control ones. Metabolites were detected and identified by GC-MS. Metabolites indicated in black were observed, while metabolites indicated in grey were not detected. The median *m/z* intensity (red line) in the box- whisker plots was used to compare the level of metabolites. (A) Represent the level of metabolites at 10 min, while (B) the level of metabolite at 60 min. Traffic light system represents different concentration of propranolol. Red, yellow and green represent exposed cells to 0.2, 0.4 and 0.6 mg/mL of propranolol respectively. Up-arrow, down-arrow and steady arrow indicate an increase, a decrease and no change in the level of metabolite respectively. The number of arrows represents the level of metabolites. Slight change (single arrow), medium change (double arrows) and high change (triple arrows).

It is clear from the above that there are changes in central metabolism in response to propranolol exposure. Therefore, we investigated whether the levels of metabolites in the central metabolic pathways of *P*. *putida* strains were significantly altered or not between control and propranolol challenged samples for each bacterial strain independently. Metabolic pathways that were changed during propranolol stress were identified utilising untargeted GC-MS analysis. A comparative summary of central metabolic pathways between control and propranolol challenged cells for 10 or 60 min in *P*. *putida* DOT-T1E (Fig 5), *P*. *putida* DOT-T1E-18 (Fig 6) and *P*. *putida* DOT-T1E-PS28 (S7 Fig) were generated and large effects were seen in amino acid biosynthesis. In total, 17 metabolites were differentially produced or consumed in the presence of 3 different concentrations of propranolol, compared to the control sample at two time points. Major metabolites that were changed significantly during propranolol stress were serine, glycine, tryptophan, phenylalanine, tyrosine, alanine, valine, leucine, citrate, fumarate, glutamine, ornithine, aspartic acid, lysine, methionine, threonine and isoleucine, and box-whisker plots of these metabolites show the changes in these metabolite levels (S8–S11 Figs).

In *P*. *putida* DOT-T1E, 10 metabolites were found to be consumed, 4 metabolites produced and 3 metabolites did not change at 10 min, while 4 metabolites were down-regulated and 13 metabolites up-regulated at 60 min. In the mutant DOT-T1E-18, 10 min following exposure to propranolol the levels of 2 metabolites increased, 8 metabolites were consumed and 7 metabolites remained constant. After 60 min following exposure to propranolol, the levels of 11 metabolites were increased, 3 metabolites were consumed and 3 remained constant. In *P*. *putida* DOT-T1E-PS28, although similar patterns in the level of metabolites was observed compared to the wild- type in the absence of propranolol, different patterns were observed in the presence of propranolol. Both mutants showed different metabolic profiles compared to the wild type and this could be due to the lack of the pump leading to over-expression of certain amino acids that are important to activate specific pumps or other metabolic pathways to cope with the stress.

Pathway analysis also revealed that glutamine and ornithine, which shows similar metabolic changes in all *P*. *putida* DOT-T1E strains, as major pathways impacted by propranolol stress. It is possible that glutamine could be being consumed by the cells in order to respond to high energy demands due to propranolol exposure. Another possible suggestion is that the decrease in the level of glutamine may be due to the biosynthesis of ornithine which could be the key stress-responsive metabolite involved to cope with stress following perturbation by propranolol. Therefore, cells may convert glutamate into ornithine instead of glutamine, resulting in a decrease in the level of glutamine. In contrast, in comparison to the wild type both mutants undergo different metabolic changes in other detected metabolites, mainly aliphatic amino acids, aromatic amino acids, and the aspartate family. This might be explained by the lack of the efflux pump in each mutant leading to the induction of certain metabolic pathways resulting in the production or consumption of certain amino acids associated with specific pumps.

## Conclusion

Here we have shown that propranolol had a measurable biological effect on all three strains of bacteria studied. The results demonstrated that the mutant *P*. *putida* DOT-T1E-18 was more sensitive to propranolol than the other strains analysed due to the lack of TtgABC pump. With respect to exposure to propranolol, data from FT-IR revealed that propranolol had an effect on protein components of the bacterial cells. The investigation of the characterization of the metabolome of *P*. *putida* DOT-T1E strains upon exposure to propranolol revealed the important role of efflux pump activity and the production of ornithine as major key elements for adaptation mechanisms. This information can be useful in bioengineering to create engineered *P*. *putida* strains or even other bacteria with superior tolerance characteristic for bioprocesses, which in turn can help to remediate simple or complex mixtures of pollutants from environment. Similar to the case where lactate tolerance was improved in an engineered strain producing ascorbic acid, a well-known reactive-oxygen species scavenger [83]. Furthermore, both screening tools and metabolic profiling in combination with multivariate statistical methods, seem ideally suited to monitoring the phenotypic responses occurring within microbial cultures under different growth conditions and subjected to abiotic stress.

## Supporting Information

S1 Fig MB-PCA scores plot of GC-MS data for the wild type and the mutants in the *absence* of propranolol. Colours represent different strains. (A) *P*. *putida* DOT-T1E is the wild type (red), (B) *P*. *putida* DOT-T1E-PS28 (green), and (C) *P*. *putida* DOT-T1E-18 (blue).
(PDF)

S2 Fig Box-whisker plots of a few selected most significant metabolites between the wild type and the mutants in the *absence* of propranolol. (A) *P*. *putida* DOT-T1E is the wild type, (B) *P*. *putida* DOT-T1E-PS28, and (C) *P*. *putida* DOT-T1E-18. Variables 9 (unknown), Variables 29 (leucine), Variables 37 (leucine^), Variables 70 (unknown), Variables 134 (à-D-glucopyranoside*), Variables 163 (D-ribonic acid/ D-glucose*), Variables 185 (á-N-acetylneuraminic acid/ D-Glucose*), Variables 188 (sucrose), and Variables 198 (á-N-acetylneuraminic acid*). ^ multiple derivatives of same compound. *multiple assignments as identification is putative only.
(PDF)

S3 Fig Schematic metabolic pathway diagram of central carbon metabolism in *P*. *putida* DOT-T1E showing the level of metabolites for both mutants compared to the wild type. Metabolites were detected and identified by GC-MS. Metabolites indicated in black were observed, while metabolites indicated in grey were not detected. The median *m/z* intensity (red line) in the box-whisker plots was used to compare the level of metabolites. Blue and brown represent the mutant DOT-T1E-PS28 and DOT-T1E-18 respectively. Up-arrow, down-arrow and steady arrow indicate an increase, a decrease and no change in the level of metabolite respectively. The number of arrows represents the level of metabolites. Slight change (single arrow) and medium change (double arrows).
(PDF)

S4 Fig Validated PC-DFA models of (A) *P*. *putida* DOT-T1E, (B) *P*. *putida* DOT-T1E-PS28, (C) *P*. *putida* DOT-T1E-18 upon 0.2, 0.4 and 0.6 mg mL^-1^ Propranolol shock. Symbols coding: control with no propranolol (circles), cells exposed to 0.2 mg mL^-1^ propranolol (squares), 0.4 mg mL^-1^ propranolol (triangles), and 0.6 mg mL^-1^ propranolol (upside down triangles). Opened symbols represent the test set while closed symbols represent the training set.
(PDF)

S5 Fig MB-PCA score plot of GC-MS data showing the effect of different concentrations on *P*. *putida* strains. Colours represent different dosage of propranolol. (D0) exposed to 0 mg/mL propranolol (blue), (D1) exposed to 0.2 mg mL^-1^ propranolol (green), and (D2) exposed to 0.4 mg mL^-1^ propranolol (pink). (D3) exposed to 0.6 mg mL^-1^ propranolol (red).
(PDF)

S6 Fig MB-PCA loading plot of GC-MS data showing the most significant metabolites in the presence of different concentrations of propranolol. Significant loadings were observed in the positive and negative sides of the plot.
(PDF)

S7 Fig Schematic metabolic diagram of central carbon metabolism in *P*. *putida* DOT-T1E-PS28 adapted to propranolol showing the level of metabolites for cells exposed to propranolol compared to the control. Metabolites were detected and identified by GC-MS. Metabolites indicated in black were observed, while metabolites indicated in grey were not detected. The median *m/z* intensity (red line) in the box- whisker plots was used to compare the level of metabolites. (A) Represent the level of metabolites at 10 min, while (B) the level of metabolite at 60 min. Traffic light system represents different concentration of propranolol. Red, yellow and green represent exposed cells to 0.2, 0.4 and 0.6 mg mL^-1^ of propranolol respectively. Up-arrow, down-arrow and steady arrow indicate an increase, a decrease and no change in the level of metabolite respectively. The number of arrows represents the level of metabolites. Slight change (single arrow), medium change (double arrows) and high change (triple arrows).
(PDF)

S8 Fig Box-whisker plots of the detected metabolites of central carbon metabolism in *P*. *putida* DOT-T1E, DOT-T1E-PS28 and DOT-T1E-18. Dashed lines separate different concentration levels of propranolol and solid line separates different strains. The label is constructed in a format of “Aac”, “A” represents strains, varies from A to C: A = *P*. *putida* DOT-T1E; B = *P*. *putida* DOT-T1E-PS28 and C = *P*. *putida* DOT-T1E-18. “a” represents 4 different concentration levels, varies from 0 to 3: 0 = control; 1 = 0.2 mg mL^-1^; 2 = 0.4 mg mL^-1^ and 3 = 0.6 mg mL^-1^ propranolol. “c” represents time points, 1 = T0 (0 min); 2 = T1 (10 min) and 3 = T2 (1 h). Such plots give a comprehensive view of how the concentration levels of the metabolite changing under each unique combination of the factors (strains, dosage of propranolol and time). Variables 14 (alanine), Variables 20 (valine), Variables 29 (leucine), and Variables 34 (isoleucine).
(PDF)

S9 Fig Box-whisker plots of the detected metabolites of central carbon metabolism in *P*. *putida* DOT-T1E, DOT-T1E-PS28 and DOT-T1E-18. Dashed lines separate different concentration levels of propranolol and solid line separates different strains. The label is constructed in a format of “Aac”, “A” represents strains, varies from A to C: A = *P*. *putida* DOT-T1E; B = *P*. *putida* DOT-T1E-PS28 and C = *P*. *putida* DOT-T1E-18. “a” represents 4 different concentration levels, varies from 0 to 3: 0 = control; 1 = 0.2 mg mL^-1^; 2 = 0.4 mg mL^-1^ and 3 = 0.6 mg mL^-1^ propranolol. “c” represents time points, 1 = T0 (0 min); 2 = T1 (10 min) and 3 = T2 (1 h). Such plots give a comprehensive view of how the concentration levels of the metabolite changing under each unique combination of the factors (strains, dosage of propranolol and time). Variables 40 (glycine), Variables 53 (threonine), Variables 54 (serine), and Variables 78 (aspartic acid).
(PDF)

S10 Fig Box-whisker plots of the detected metabolites of central carbon metabolism in *P*. *putida* DOT-T1E, DOT-T1E-PS28 and DOT-T1E-18. Dashed lines separate different concentration levels of propranolol and solid line separates different strains. The label is constructed in a format of “Aac”, “A” represents strains, varies from A to C: A = *P*. *putida* DOT-T1E; B = *P*. *putida* DOT-T1E-PS28 and C = *P*. *putida* DOT-T1E-18. “a” represents 4 different concentration levels, varies from 0 to 3: 0 = control; 1 = 0.2 mg mL^-1^; 2 = 0.4 mg mL^-1^ and 3 = 0.6 mg mL^-1^ propranolol. “c” represents time points, 1 = T0 (0 min); 2 = T1 (10 min) and 3 = T2 (1 h). Such plots give a comprehensive view of how the concentration levels of the metabolite changing under each unique combination of the factors (strains, dosage of propranolol and time). Variables 81 (methionine), Variables 88 (glutamine), Variables 95 (phenylalanine), and Variables 103 (fumarate).
(PDF)

S11 Fig Box-whisker plots of the detected metabolites of central carbon metabolism in *P*. *putida* DOT-T1E, DOT-T1E-PS28 and DOT-T1E-18. Dashed lines separate different concentration levels of propranolol and solid line separates different strains. The label is constructed in a format of “Aac”, “A” represents strains, varies from A to C: A = *P*. *putida* DOT-T1E; B = *P*. *putida* DOT-T1E-PS28 and C = *P*. *putida* DOT-T1E-18. “a” represents 4 different concentration levels, varies from 0 to 3: 0 = control; 1 = 0.2 mg mL^-1^; 2 = 0.4 mg mL^-1^ and 3 = 0.6 mg mL^-1^ propranolol. “c” represents time points, 1 = T0 (0 min); 2 = T1 (10 min) and 3 = T2 (1 h). Such plots give a comprehensive view of how the concentration levels of the metabolite changing under each unique combination of the factors (strains, dosage of propranolol and time). Variables 109 (citrate), Variables 119 (lysine), Variables 135 (tyrosine), and Variables 177 (tryptophan).
(PDF)

S1 Table The level of metabolites for both mutants compared to the wild type in the central carbon metabolism in *P*. *putida* DOT-T1E. Metabolites were detected and identified by GC-MS.
(PDF)

S2 Table Results from the propranolol MIC experiments using *P*. *putida* DOT-T1E, DOT-T1E-PS28 and DOT-T1E-18. Culture growth was observed after overnight incubation.
(PDF)

S3 Table Viability of *P*. *putida* cells 1 h later after exposure to propranolol. (PDF)
