# Supplementary figures and images for: Metabolomics Analysis Reveals the Participation of Efflux Pumps and Ornithine in the Response of Pseudomonas putida DOT-T1E Cells to Challenge with Propranolol
Source: PLoS One. 2016 Jun 22;11(6):e0156509. doi: 10.1371/journal.pone.0156509 (PMC4917112; doi:10.1371/journal.pone.0156509)

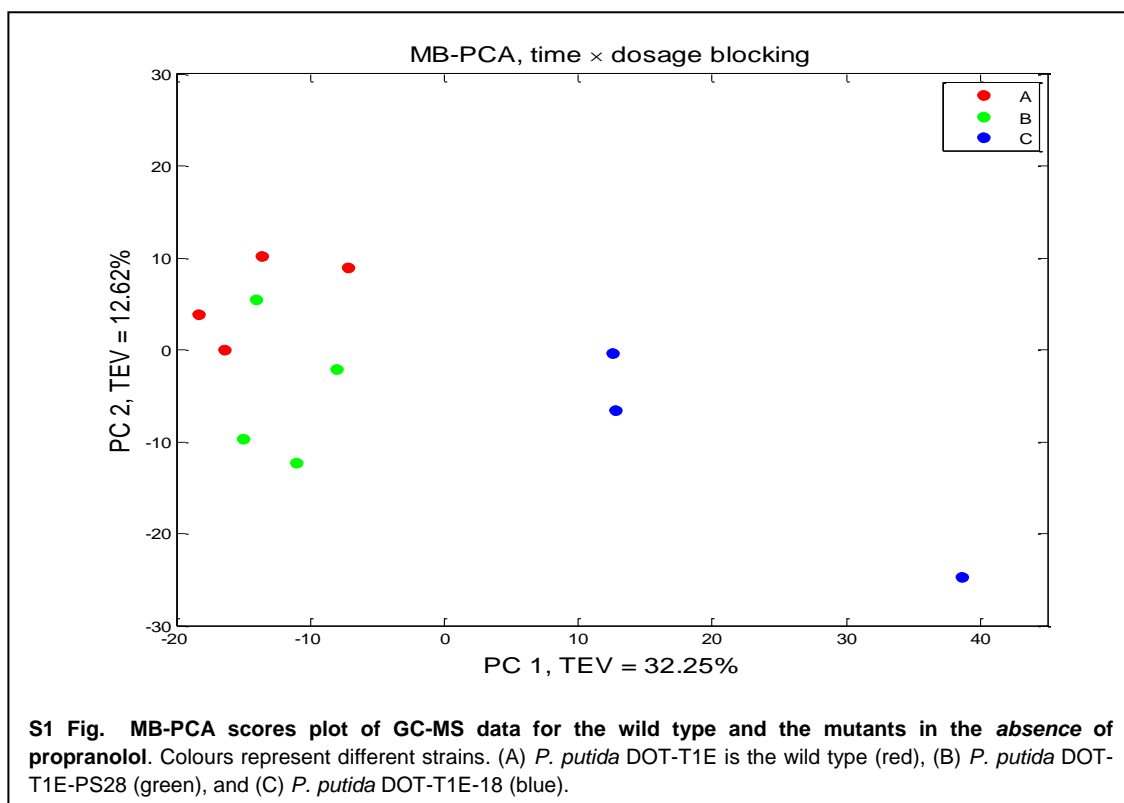

Supplement: S1 Fig — Colours represent different strains. (A) P. putida DOT-T1E is the wild type (red), (B) P. putida DOT-T1E-PS28 (green), and (C) P. putida DOT-T1E-18 (blue). (PDF) [file pone.0156509.s001.pdf]

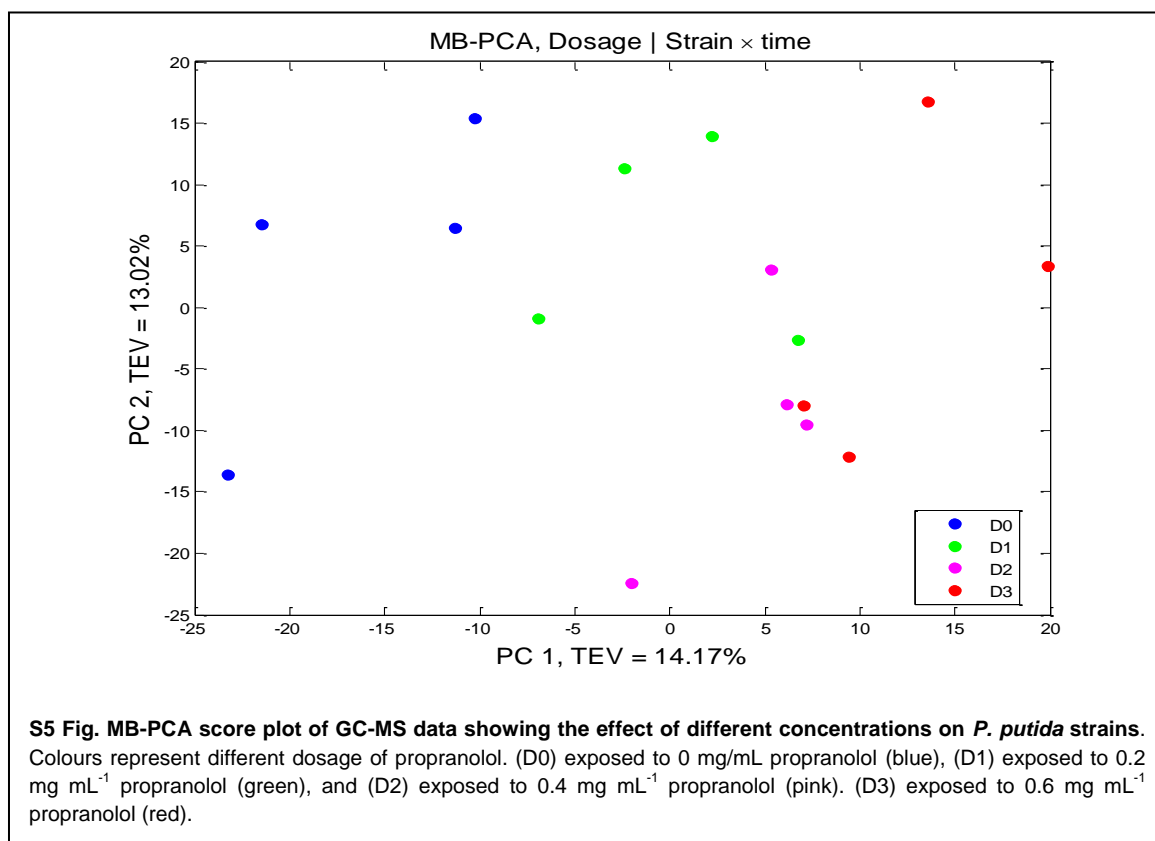

Supplement: S5 Fig — Colours represent different dosage of propranolol. (D0) exposed to 0 mg/mL propranolol (blue), (D1) exposed to 0.2 mg mL-1 propranolol (green), and (D2) exposed to 0.4 mg mL-1 propranolol (pink). (D3) exposed to 0.6 mg mL-1 propranolol (red). (PDF) [file pone.0156509.s005.pdf]

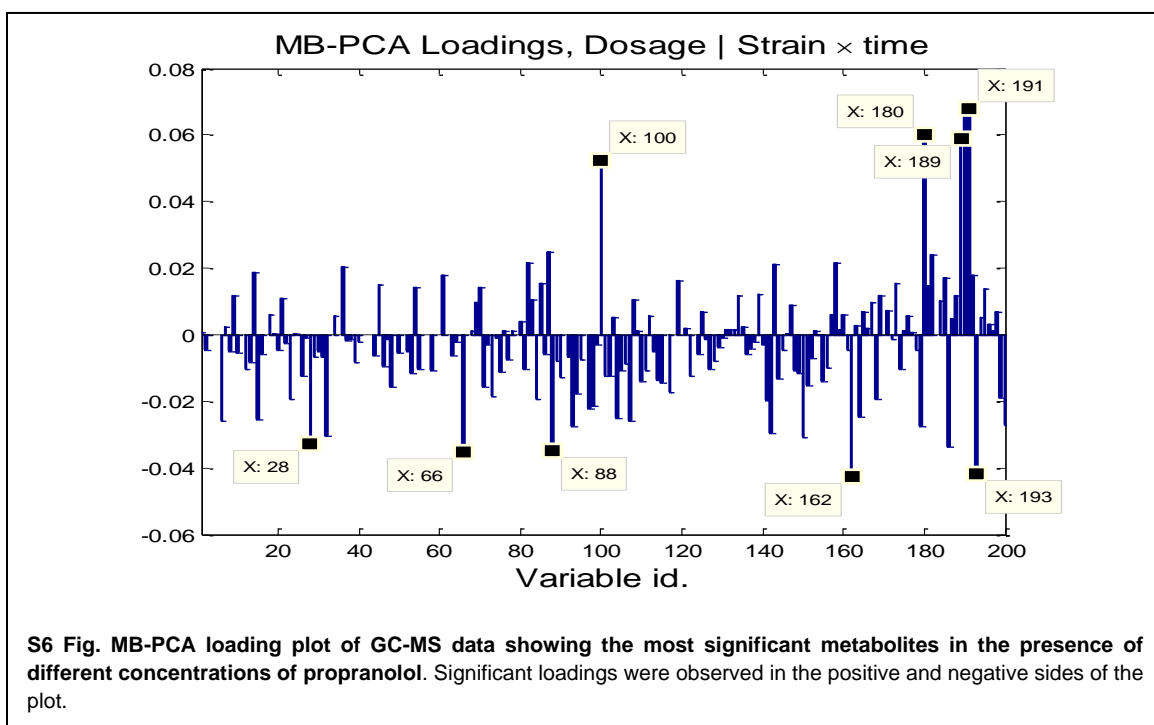

Supplement: S6 Fig — Significant loadings were observed in the positive and negative sides of the plot. (PDF) [file pone.0156509.s006.pdf]

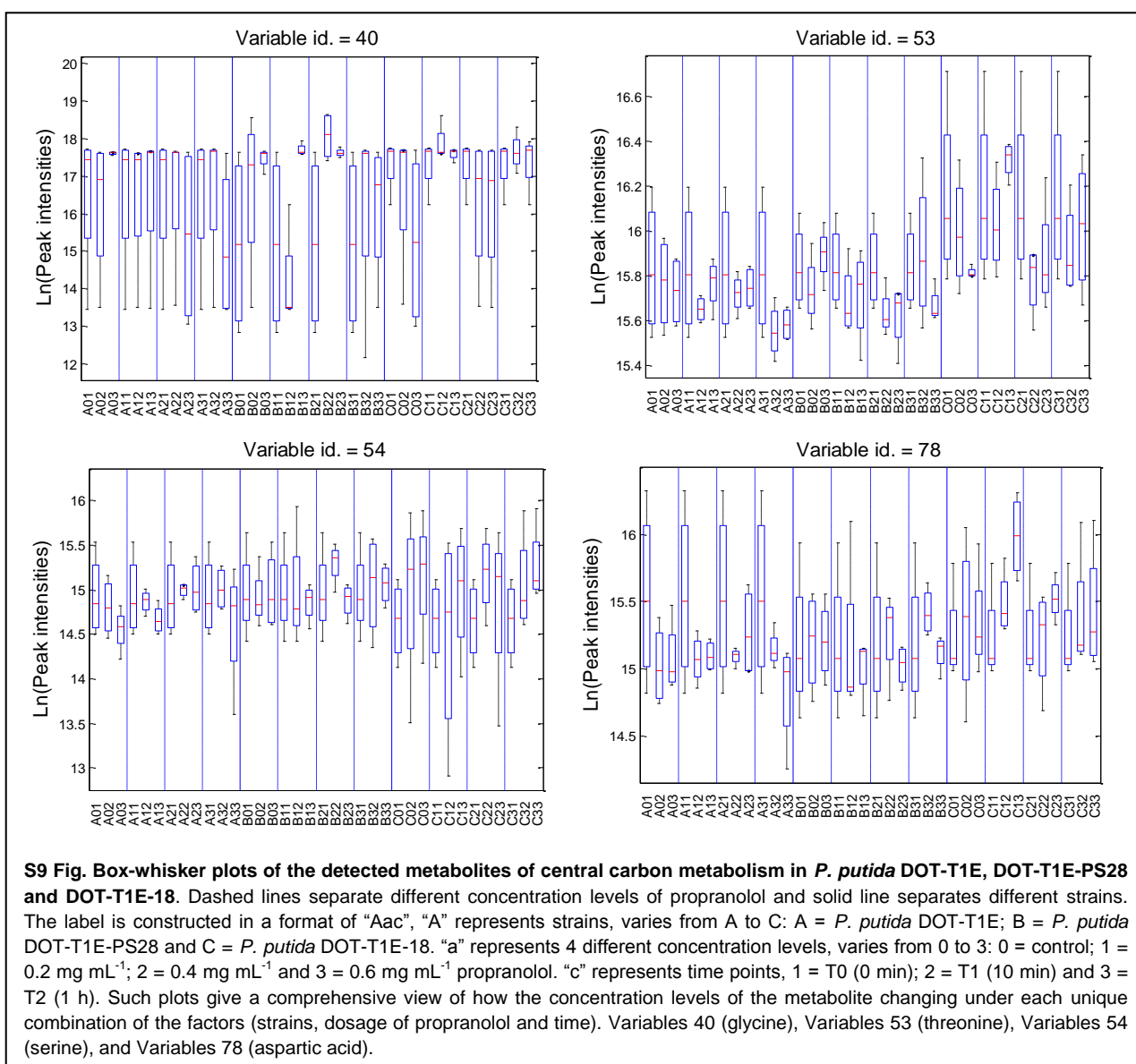

Supplement: S9 Fig — Dashed lines separate different concentration levels of propranolol and solid line separates different strains. The label is constructed in a format of “Aac”, “A” represents strains, varies from A to C: A = P. putida DOT-T1E; B = P. putida DOT-T1E-PS28 and C = P. putida DOT-T1E-18. “a” represents 4 different concentration levels, varies from 0 to 3: 0 = control; 1 = 0.2 mg mL-1; 2 = 0.4 mg mL-1 and 3 = 0.6 mg mL-1 propranolol. “c” represents time points, 1 = T0 (0 min); 2 = T1 (10 min) and 3 = T2 (1 h). Such plots give a comprehensive view of how the concentration levels of the metabolite changing under each unique combination of the factors (strains, dosage of propranolol and time). Variables 40 (glycine), Variables 53 (threonine), Variables 54 (serine), and Variables 78 (aspartic acid). (PDF) [file pone.0156509.s009.pdf]
