# Supplementary material for: Metabolomics Analysis Reveals the Participation of Efflux Pumps and Ornithine in the Response of Pseudomonas putida DOT-T1E Cells to Challenge with Propranolol
Source: PLoS One. 2016 Jun 22;11(6):e0156509. doi: 10.1371/journal.pone.0156509 (PMC4917112; doi:10.1371/journal.pone.0156509)
Supplement: S2 Fig — (A) P. putida DOT-T1E is the wild type, (B) P. putida DOT-T1E-PS28, and (C) P. putida DOT-T1E-18. Variables 9 (unknown), Variables 29 (leucine), Variables 37 (leucine^), Variables 70 (unknown), Variables 134 (à-D-glucopyranoside*), Variables 163 (D-ribonic acid/ D-glucose*), Variables 185 (á-N-acetylneuraminic acid/ D-Glucose*), Variables 188 (sucrose), and Variables 198 (á-N-acetylneuraminic acid*). ^ multiple derivatives of same compound. *multiple assignments as identification is putative only. (PDF) [file pone.0156509.s002.pdf]

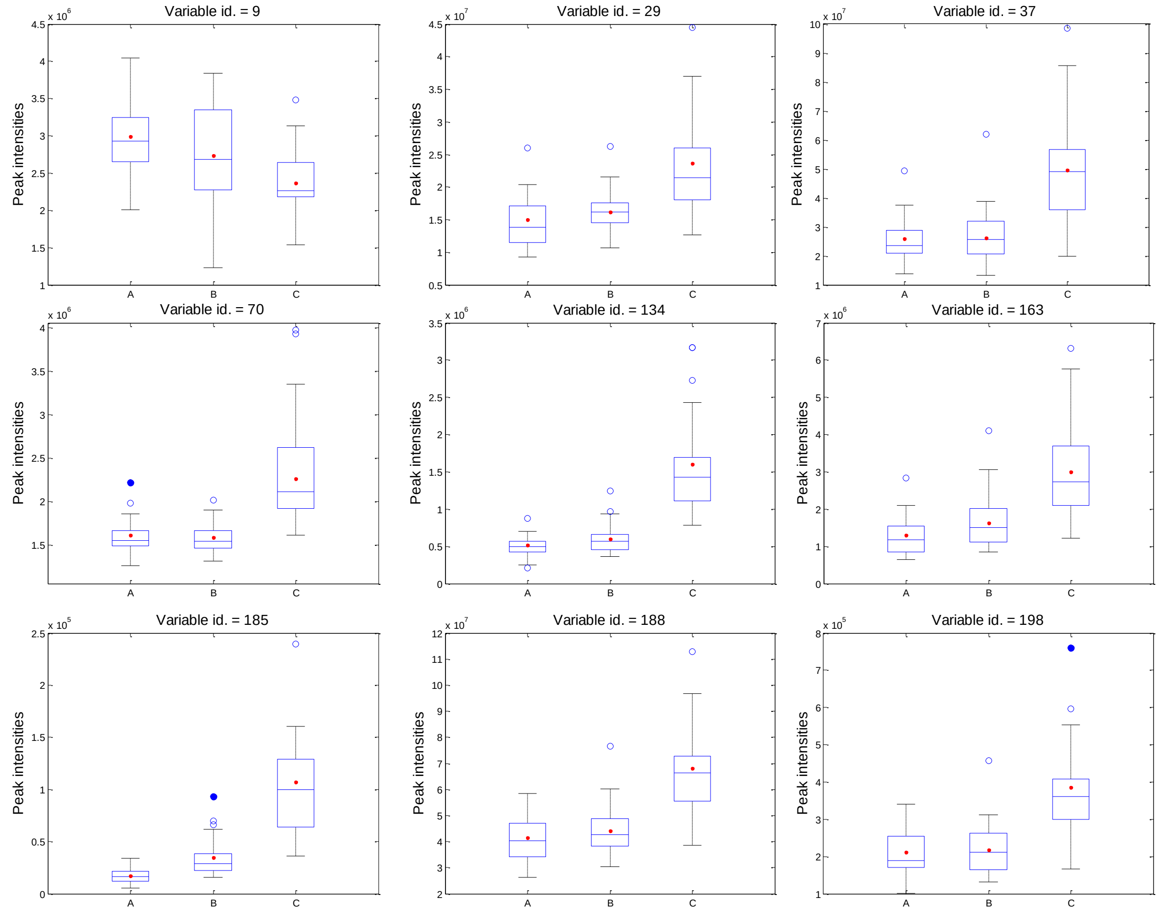

S2 Fig. Box-whisker plots of a few selected most significant metabolites between the wild type and the mutants in the *absence* of propranolol. (A) *P. putida* DOT-T1E is the wild type, (B) *P. putida* DOT-T1E-PS28, and (C) *P. putida* DOT-T1E-18. Variables 9 (unknown), Variables 29 (leucine), Variables 37 (leucine^), Variables 70 (unknown), Variables 134 (à-D-glucopyranoside*), Variables 163 (D-ribonic acid/ D-glucose*), Variables 185 (á-N-acetylneuraminic acid/ D-Glucose*), Variables 188 (sucrose), and Variables 198 (á-N-acetylneuraminic acid*).

^ multiple derivatives of same compound *multiple assignments as identification is putative only
