# Supplementary material for: Metabolomics Analysis Reveals the Participation of Efflux Pumps and Ornithine in the Response of Pseudomonas putida DOT-T1E Cells to Challenge with Propranolol
Source: PLoS One. 2016 Jun 22;11(6):e0156509. doi: 10.1371/journal.pone.0156509 (PMC4917112; doi:10.1371/journal.pone.0156509)
Supplement: S3 Fig — Metabolites were detected and identified by GC-MS. Metabolites indicated in black were observed, while metabolites indicated in grey were not detected. The median m/z intensity (red line) in the box-whisker plots was used to compare the level of metabolites. Blue and brown represent the mutant DOT-T1E-PS28 and DOT-T1E-18 respectively. Up-arrow, down-arrow and steady arrow indicate an increase, a decrease and no change in the level of metabolite respectively. The number of arrows represents the level of metabolites. Slight change (single arrow) and medium change (double arrows). (PDF) [file pone.0156509.s003.pdf]

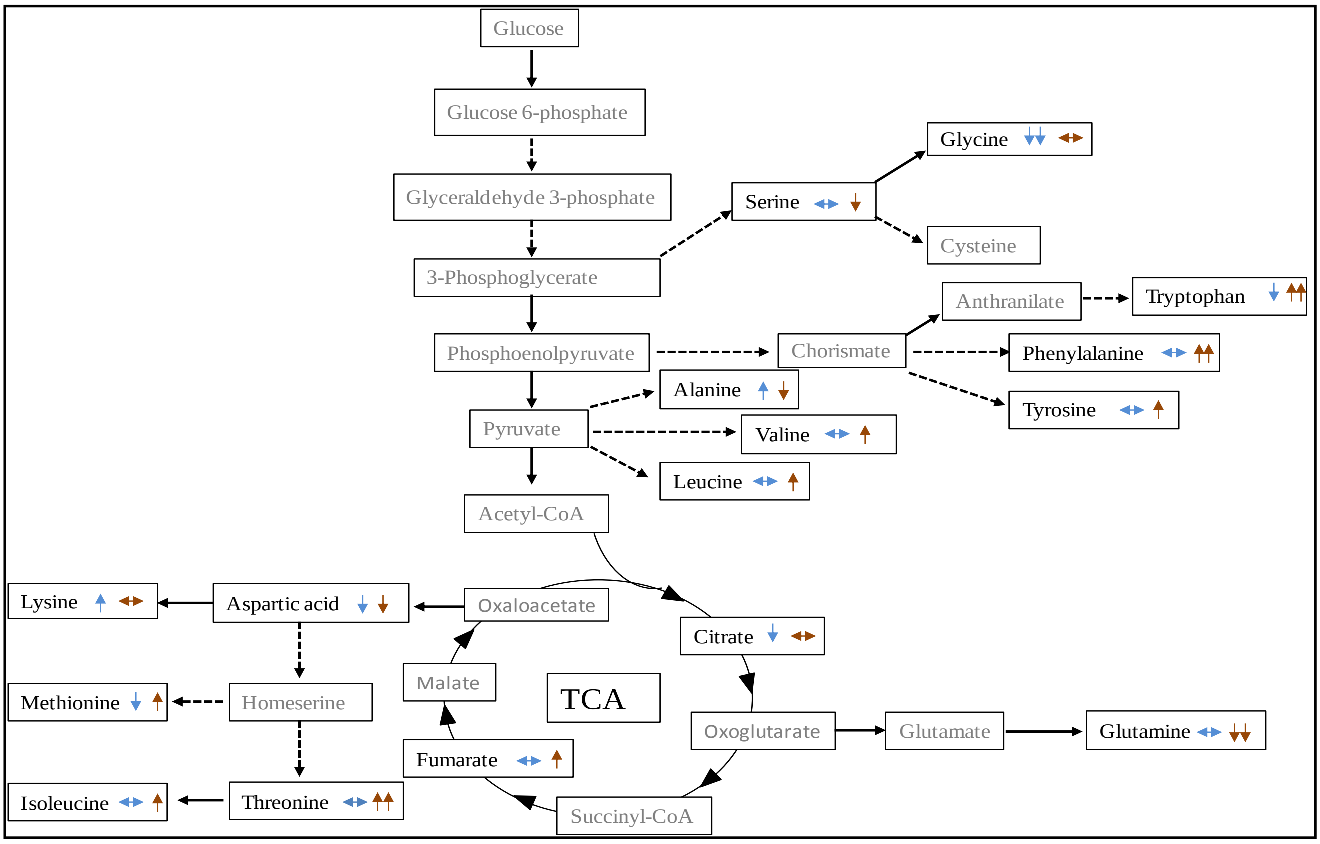

S3 Fig. Schematic metabolic pathway diagram of central carbon metabolism in *P. putida* DOT-T1E showing the level of metabolites for both mutants compared to the wild type. Metabolites were detected and identified by GC-MS. Metabolites indicated in black were observed, while metabolites indicated in grey were not detected. The median *m/z* intensity (red line) in the box-whisker plots was used to compare the level of metabolites. Blue and brown represent the mutant DOT-T1E-PS28 and DOT-T1E-18 respectively. Up-arrow, down-arrow and steady arrow indicate an increase, a decrease and no change in the level of metabolite respectively. The number of arrows represents the level of metabolites. Slight change (single arrow) and medium change (double arrows).
