# Supplementary material for: Metabolomics Analysis Reveals the Participation of Efflux Pumps and Ornithine in the Response of Pseudomonas putida DOT-T1E Cells to Challenge with Propranolol
Source: PLoS One. 2016 Jun 22;11(6):e0156509. doi: 10.1371/journal.pone.0156509 (PMC4917112; doi:10.1371/journal.pone.0156509)
Supplement: S4 Fig — Symbols coding: control with no propranolol (circles), cells exposed to 0.2 mg mL-1 propranolol (squares), 0.4 mg mL-1 propranolol (triangles), and 0.6 mg mL-1 propranolol (upside down triangles). Opened symbols represent the test set while closed symbols represent the training set. (PDF) [file pone.0156509.s004.pdf]

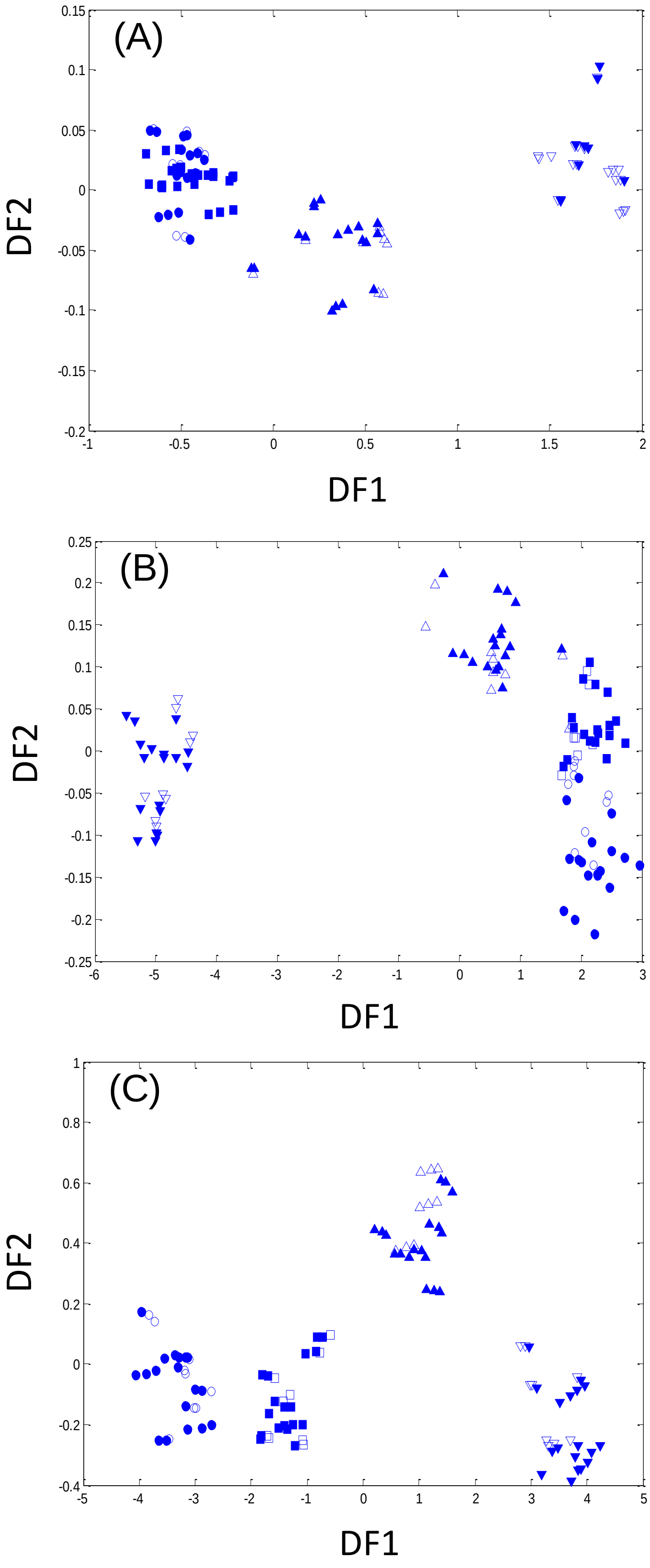

S4 Fig. Validated PC-DFA models of (A) *P. putida* DOT-T1E, (B) *P. putida* DOT-T1E-PS28, (C) *P. putida* DOT-T1E-18 upon 0.2, 0.4 and 0.6 mg mL^{-1} Propranolol shock. Symbols coding: control with no propranolol (circles), cells exposed to 0.2 mg mL^{-1} propranolol (squares), 0.4 mg mL^{-1} propranolol (triangles), and 0.6 mg mL^{-1} propranolol (upside down triangles). Opened symbols represent the test set while closed symbols represent the training set.
