# Supplementary material for: Metabolomics Analysis Reveals the Participation of Efflux Pumps and Ornithine in the Response of Pseudomonas putida DOT-T1E Cells to Challenge with Propranolol
Source: PLoS One. 2016 Jun 22;11(6):e0156509. doi: 10.1371/journal.pone.0156509 (PMC4917112; doi:10.1371/journal.pone.0156509)
Supplement: S7 Fig — Metabolites were detected and identified by GC-MS. Metabolites indicated in black were observed, while metabolites indicated in grey were not detected. The median m/z intensity (red line) in the box- whisker plots was used to compare the level of metabolites. (A) Represent the level of metabolites at 10 min, while (B) the level of metabolite at 60 min. Traffic light system represents different concentration of propranolol. Red, yellow and green represent exposed cells to 0.2, 0.4 and 0.6 mg mL-1 of propranolol respectively. Up-arrow, down-arrow and steady arrow indicate an increase, a decrease and no change in the level of metabolite respectively. The number of arrows represents the level of metabolites. Slight change (single arrow), medium change (double arrows) and high change (triple arrows). (PDF) [file pone.0156509.s007.pdf]

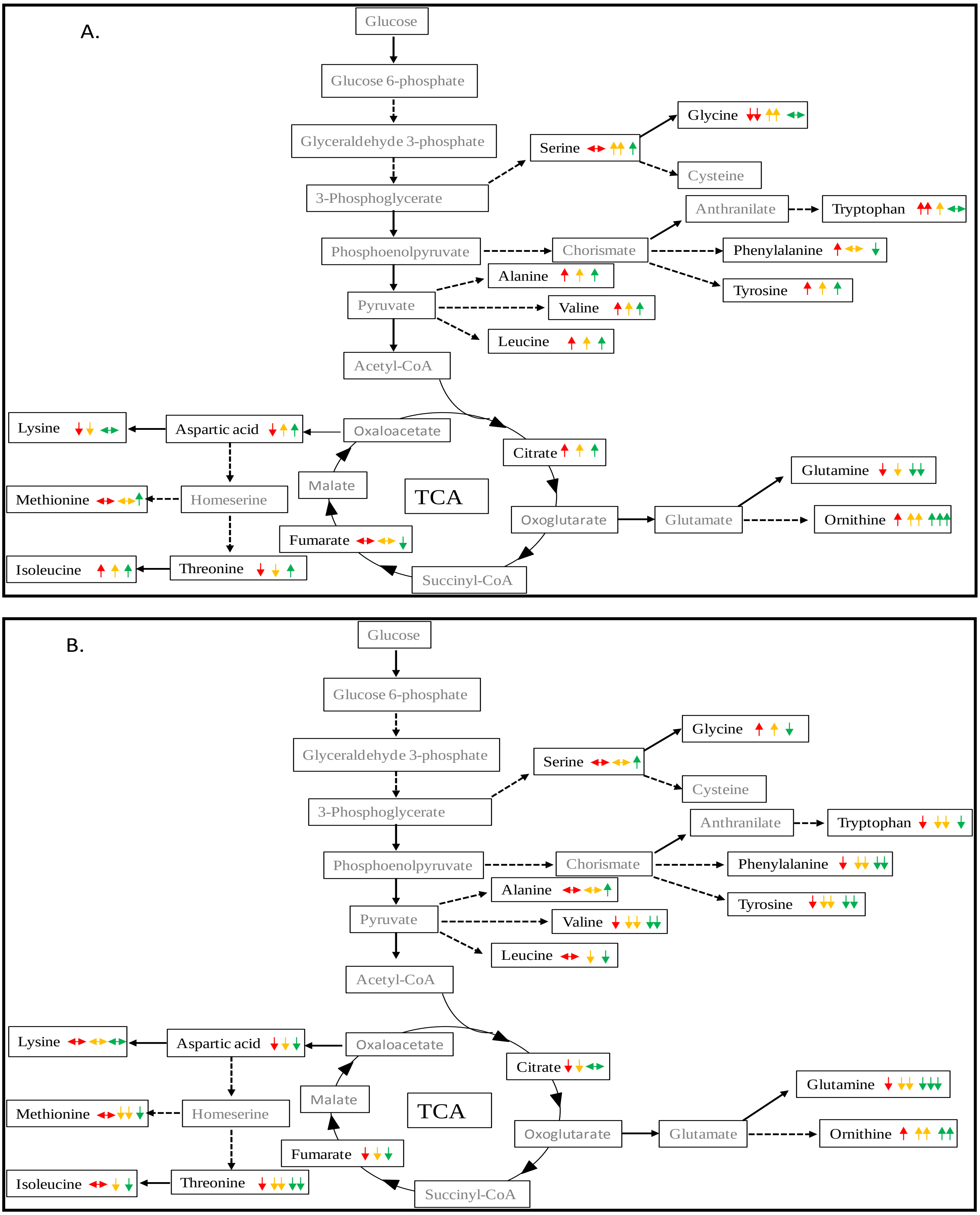

S7 Fig. Schematic metabolic diagram of central carbon metabolism in *P. putida* DOT-T1E-PS28 adapted to propranolol showing the level of metabolites for cells exposed to propranolol compared to the control. Metabolites were detected and identified by GC-MS. Metabolites indicated in black were observed, while metabolites indicated in grey were not detected. The median *m/z* intensity (red line) in the box- whisker plots was used to compare the level of metabolites. (A) Represent the level of metabolites at 10 min, while (B) the level of metabolite at 60 min. Traffic light system represents different concentration of propranolol. Red, yellow and green represent exposed cells to 0.2, 0.4 and 0.6 mg mL^{-1} of propranolol respectively. Up-arrow, down-arrow and steady arrow indicate an increase, a decrease and no change in the level of metabolite respectively. The number of arrows represents the level of metabolites. Slight change (single arrow), medium change (double arrows) and high change (triple arrows).
