# Supplementary material for: Metabolomics Analysis Reveals the Participation of Efflux Pumps and Ornithine in the Response of Pseudomonas putida DOT-T1E Cells to Challenge with Propranolol
Source: PLoS One. 2016 Jun 22;11(6):e0156509. doi: 10.1371/journal.pone.0156509 (PMC4917112; doi:10.1371/journal.pone.0156509)
Supplement: S10 Fig — Dashed lines separate different concentration levels of propranolol and solid line separates different strains. The label is constructed in a format of “Aac”, “A” represents strains, varies from A to C: A = P. putida DOT-T1E; B = P. putida DOT-T1E-PS28 and C = P. putida DOT-T1E-18. “a” represents 4 different concentration levels, varies from 0 to 3: 0 = control; 1 = 0.2 mg mL-1; 2 = 0.4 mg mL-1 and 3 = 0.6 mg mL-1 propranolol. “c” represents time points, 1 = T0 (0 min); 2 = T1 (10 min) and 3 = T2 (1 h). Such plots give a comprehensive view of how the concentration levels of the metabolite changing under each unique combination of the factors (strains, dosage of propranolol and time). Variables 81 (methionine), Variables 88 (glutamine), Variables 95 (phenylalanine), and Variables 103 (fumarate). (PDF) [file pone.0156509.s010.pdf]

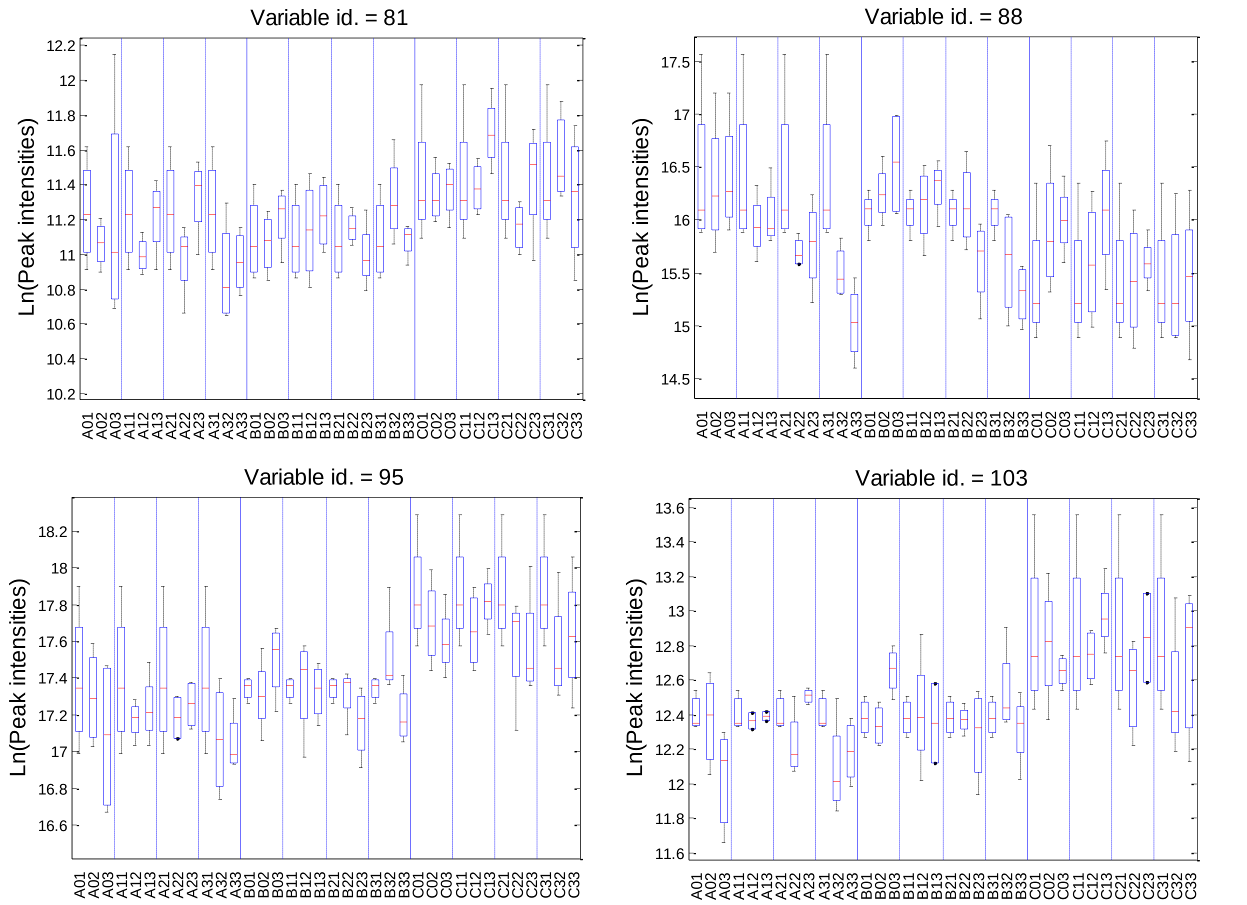

S10 Fig. Box-whisker plots of the detected metabolites of central carbon metabolism in *P. putida* DOT-T1E, DOT-T1E-PS28 and DOT-T1E-18. Dashed lines separate different concentration levels of propranolol and solid line separates different strains. The label is constructed in a format of "Aac", "A" represents strains, varies from A to C: A = *P. putida* DOT-T1E; B = *P. putida* DOT-T1E-PS28 and C = *P. putida* DOT-T1E-18. "a" represents 4 different concentration levels, varies from 0 to 3: 0 = control; 1 = 0.2 mg mL^{-1}; 2 = 0.4 mg mL^{-1} and 3 = 0.6 mg mL^{-1} propranolol. "c" represents time points, 1 = T0 (0 min); 2 = T1 (10 min) and 3 = T2 (1 h). Such plots give a comprehensive view of how the concentration levels of the metabolite changing under each unique combination of the factors (strains, dosage of propranolol and time). Variables 81 (methionine), Variables 88 (glutamine), Variables 95 (phenylalanine), and Variables 103 (fumarate).
