# Supplementary material for: Metabolomics Analysis Reveals the Participation of Efflux Pumps and Ornithine in the Response of Pseudomonas putida DOT-T1E Cells to Challenge with Propranolol
Source: PLoS One. 2016 Jun 22;11(6):e0156509. doi: 10.1371/journal.pone.0156509 (PMC4917112; doi:10.1371/journal.pone.0156509)
Supplement: S1 Table — Metabolites were detected and identified by GC-MS. (PDF) [file pone.0156509.s012.pdf]

S1 Table The level of metabolites for both mutants compared to the wild type in the central carbon metabolism in *P. putida* DOT-T1E. Metabolites were detected and identified by GC-MS.

Variable No	Metabolites ID	The level of metabolites*	
		M2 *vs*. M1	M3 *vs*. M1
14	Alanine	up	Down
20	Valine	n/c	Up
29	Leucine	n/c	Up
34	Isoleucine	n/c	Up
40	Glycine	down*	n/c
53	Threonine	n/c	up*
54	Serine	n/c	Down
78	Aspartic Acid	down	Down
81	Methionine	down	Up
88	Glutamine	n/c	down*
95	Phenylalanine	n/c	up*
103	Fumarate	n/c	Up
109	Citrate	down	n/c
119	Lysine	up	n/c
135	Tyrosine	n/c	Up
177	Tryptophan	down	up*

*See explanation in Figure S3, M1: DOT-T1E, M2: DOT-T1E-PS28, M3: DOT-T1E-18, n/c: no change
