# Supplementary material for: Metabolomics Analysis Reveals the Participation of Efflux Pumps and Ornithine in the Response of Pseudomonas putida DOT-T1E Cells to Challenge with Propranolol
Source: PLoS One. 2016 Jun 22;11(6):e0156509. doi: 10.1371/journal.pone.0156509 (PMC4917112; doi:10.1371/journal.pone.0156509)
Supplement: S2 Table — Culture growth was observed after overnight incubation. (PDF) [file pone.0156509.s013.pdf]

S2 Table Results from the propranolol MIC experiments using *P. putida* DOT-T1E, DOT-T1E-PS28 and DOT-T1E-18. Culture growth was observed after overnight incubation.

P. putida strains	Propranolol concentration (mg mL^{-1})	Growth (+/-)*
DOT-T1E	0	+
	0.5	+
	0.7	+
	0.8	+
	1.2	± or +
	1.4	±
	1.5	-
	2	-
	2.5	-
DOT-T1E-PS28	0	+
	0.5	+
	0.7	+
	0.8	+
	1.2	± or +
	1.4	±
	1.5	-
	2	-
	2.5	-
DOT-T1E-18	0	+
	0.5	± or +
	0.7	±
	0.8	-
	1.2	-
	1.4	-
	1.5	-
	2	-
	2.5	-

*(+) indicates growth, (±) slight growth, and (-) no growth
