# Supplementary material for: Metabolomics Analysis Reveals the Participation of Efflux Pumps and Ornithine in the Response of Pseudomonas putida DOT-T1E Cells to Challenge with Propranolol
Source: PLoS One. 2016 Jun 22;11(6):e0156509. doi: 10.1371/journal.pone.0156509 (PMC4917112; doi:10.1371/journal.pone.0156509)
Supplement: S3 Table — (PDF) [file pone.0156509.s014.pdf]

S3 Table Viability of *P. putida* cells 1 h later after exposure to propranolol.

P. putida strains	Propranolol (mg mL^{-1})	Green/Red ratio*	Bacterial viability (%)
DOT-T1E	0	3.21	96.17
	0.2	2.70	73.07
	0.4	2.57	66.93
	0.6	1.83	33.03
DOT-T1E-PS28	0	3.19	95.45
	0.2	2.54	65.46
	0.4	2.10	45.19
	0.6	1.67	25.80
DOT-T1E-18	0	2.52	64.65
	0.2	2.23	51.36
	0.4	1.53	19.09
	0.6	1.26	6.89

* *Bac*Light bacterial viability assay where Green indicates live cells and Red indicates dead cells
